# Reprogramming the pancreatic ductal adenocarcinoma microenvironment: a novel integrin-targeted cytotoxin, ProAgio, potentiates chemotherapy

**DOI:** 10.1186/s12943-026-02725-3

**Published:** 2026-07-03

**Authors:** Ganji Purnachandra Nagaraju, Sujith Sarvesh, Ravi Chakra Turaga, Dhana Sekhar Reddy Bandi, Ganesh Satyanarayana, Falguni Mishra, Malvika Sharma, Mehmet Akce, Zhi-Ren Liu, Bassel F. El-Rayes

**Affiliations:** 1 Division of Hematology and Oncology, Heersink School of Medicine, University of Alabama at Birmingham, Birmingham, AL 35233 USA; 2 Division of Medical Oncology, Moores Cancer Center, UC San Diego, La Jolla, CA 92037 USA; 3 Caris Life Sciences, Tempe, AZ 85281 USA; 4 Department of Biology, Georgia State University, Atlanta, GA 30303 USA; 5 Emory School of Medicine, Emory University, Atlanta, GA 30322 USA; 6 Program in Molecular Pharmacology, Department of Medicine, Memorial Sloan Kettering Cancer Center, New York, NY 10065 USA

**Keywords:** Pancreatic Ductal Adenocarcinoma, Microenvironment, single cell RNA sequence, ProAgio, Chemotherapy

## Abstract

**Background:**

Pancreatic ductal adenocarcinoma (PDAC) growth and metastasis are influenced by the tumor microenvironment (TME), which includes immune cells, endothelial cells, macrophages, and cancer-associated fibroblasts (CAFs). Since the novel integrin-targeted cytotoxin ProAgio can inhibit activated CAFs and endothelial cells, we investigated its combination with standard of care chemotherapies in genetically engineered mouse (GEM) and orthotopic murine models of PDAC.

**Methods:**

We established metastatic murine KPC-Ganji & Bassel-Luc (mKPC-GB-Luc) cell lines and validated them using bulk RNA sequencing. Two in vivo orthotopic mouse model were used to evaluate ProAgio with chemotherapy. mKPC-GB-Luc was used to evaluate the 5FU, oxaliplatin, and irinotecan (FOI combination), and KPC-ML1-Luc was used to evaluate the gemcitabine, nab-paclitaxel (GPTx combination). Immunohistochemistry was used to measure integrin β3, E-cadherin, and HIF-1α. Hypoxia was evaluated using pimonidazole. Stem cells, CAFs, and immune cell subtypes were quantified by flow cytometry. We evaluated the combination of ProAgio plus gemcitabine in a KPC genetically engineered mouse model (KPC GEM). Two sets of KPC GEM were developed. The first set was used for a survival study. The second set was terminated early and tumors were used for single-cell RNA seq and sequential multiplex immunofluorescence (COMET).

**Results:**

Compared with the chemo regimen (GPTx or FOI), ProAgio, or sham, the combination of ProAgio plus chemo significantly modulated the TME, reduced tumor weight, reduced hypoxia, and eliminated metastasis to the lungs and liver. The combination of ProAgio and gemcitabine increased overall survival in KPC GEM mice compared with either treatment alone. Single-cell RNA sequencing, flow cytometry, immunofluorescence, immunohistochemistry, and COMET analyses demonstrated that ProAgio plus chemotherapy reprogrammed the PDAC-TME by changing activated CAFs toward a qCAFs (quiescent CAFs), macrophages from protumor to proinflammatory polarization, and activating natural killer (NK) cells, CD4⁺, and CD8⁺ T cells. Combination therapy inhibited PDAC stemness. ProAgio-treated CAFs in vitro exhibited reduced secretion of glycine and cysteine, vital metabolites that support stemness and tumor progression.

**Conclusions:**

These results confirm the novel mechanism of action of ProAgio, which includes reducing hypoxia and modulating TME. The current data provide evidence for potentiation of chemotherapy efficacy by ProAgio.

**Supplementary Information:**

The online version contains supplementary material available at https://doi.org/10.1186/s12943-026-02725-3.

## Introduction

Pancreatic ductal adenocarcinoma (PDAC) is a very aggressive disease with a current 5-year survival rate of less than 15%. The poor outcome in PDAC is due to a lack of early diagnosis and a tumor microenvironment (TME) that promotes immune evasion, chemoresistance, and tumor progression [1, 2]. Cancer-associated fibroblasts (CAFs) play a key role in shaping the extracellular matrix (ECM) and the desmoplastic stroma [2]. The stroma and aberrant angiogenesis facilitate immune evasion, tumor growth, and resistance by creating a hypoxic environment and impeding the delivery of chemotherapy [3]. A main driver of this TME is integrin-expressing activated CAFs and endothelial cells [4].

Recent literature has shown that integrins play a key role in the progression and metastasis of PDAC [5, 6]. Integrins are transmembrane heterodimeric proteins composed of α and β subunits that transmit signals bidirectionally between the intracellular cytoskeleton and the extracellular environment [7]. The integrin αvβ3 is highly expressed in stromal cells, including activated CAFs and endothelial cells [8]. The αvβ3 receptor binds to the ECM complex. It is functionally involved in the formation of new aberrant blood vessels and in the adhesion, migration, differentiation, and survival of cancer cells [8]. Integrin αvβ3 also activates intracellular signaling pathways, including those involving FAK, Src, and EGFR [9]. STAT3, a transcriptional factor, promotes ITGB3 expression, contributing to PDAC initiation and metastasis [10]. These essential pathways contribute to PDAC cell growth, ECM remodeling, and metastatic potential.

Due to its minimal expression in normal tissue and high expression in tumor tissue, especially in activated CAFs and endothelial cells, the integrin αvβ3 complex represents a compelling therapeutic target in PDAC [11]. Recently, our group developed a rationally engineered protein, ProAgio that is a cytotoxin targeted to cells that express integrin αvβ3 [12, 13]. ProAgio selectively binds to the non-catalytic domain of integrin β3 and induces apoptosis by activating caspase-8, thereby triggering integrin-mediated death, offering a novel approach to targeting PDAC-TME without affecting healthy cells [11]. Recent investigations have shown that ProAgio therapy leads to normalization of vasculature and depletion of stroma [14]. Furthermore, ProAgio potentiates gemcitabine delivery and enhances anti-tumor activity [11]. Based on these results, an ongoing trial is evaluating ProAgio in combination with gemcitabine and nab-paclitaxel in PDAC (NCT 061820072). Preliminary results from the dose-escalation phase suggest that the combination is safe.

Continued development of ProAgio in PDAC requires elucidation of several key preclinical questions. First, does ProAgio potentiate the effects of other chemotherapy regimens, specifically FOLFIRINOX? With the evidence recently published by our group on the differences in TME between primary and metastatic PDAC [15, 16], does ProAgio affect metastasis or only the primary pancreatic lesion? Does the effect of ProAgio on angiogenesis alter tumor hypoxia? With the development of our understanding of CAF subclassification and biology, how does ProAgio modulate CAFs, endothelial cells and ultimately the TME? What are the effects of reprogramming CAFs on the immune TME in PDAC? This question is important to advancing our understanding of how to enhance the clinical utility of immune therapy in PDAC. The current study aims to address these questions, which will affect the development of ProAgio, the understanding of TME biology, and the development of immune therapy in PDAC.

## Materials and methods

Submitted as a supplementary file.

## Results

### Establishment of metastatic KPC-Ganji & Bassel-Luc (mKPC-GB-Luc) cell lines

**Fig. 1:**
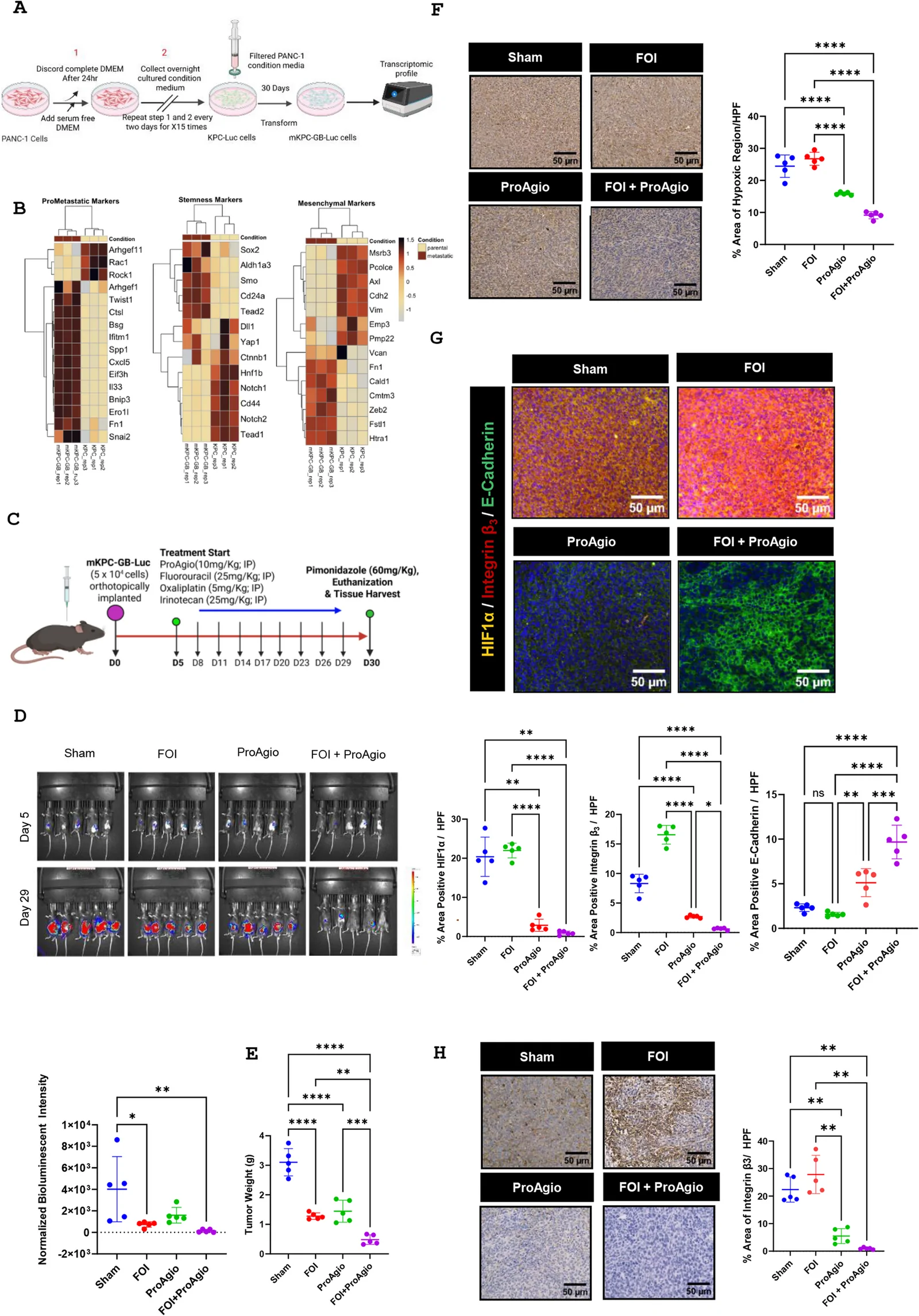
Generation of mKPC-GB-Luc cells and their validation. The effects of the combination of chemotherapy plus ProAgio in the mKPC-GB-Luc orthotopic mouse model. **A** Schema depicting experimental design for generating metastatic KPC-Ganji & Bassel-Luc (mKPC-GB-Luc) cells. **B** Bulk RNA-seq analysis reveals that prometastatic, stemness, and mesenchymal markers are upregulated in mKPC-GB-Luc compared with the parental cell line. **C **KPC schema depicting experimental design of 5-fluorouracil (5-FU), oxaliplatin, and irinotecan (FOI), and ProAgio in the mKPC-GB-Luc orthotopic murine model. **D** Bioluminescent images of mice with engrafted tumors on Day 5 and Day 29 before euthanizing with quantification of bioluminescent signal as an indicator of tumor growth in vivo (*n*=5 per treatment group). **E** Ex vivo tumor size quantification at termination of the study. **F** Representative immunohistochemistry images of pimonidazole (Hypoxprobe) along with quantification across treatment groups. **G** Representative images of immunohistochemical analysis using fluorescence probes for HIF1α, Integrinβ3, and E-Cadherin across the treatment groups. **H** Representative immunohistochemistry images of integrin β3, along with quantification across treatment groups. Scale bars 50 µm. Data is represented as mean ± S.D. A one-way ANOVA test was used to analyze the data across treatment groups, where the data were normal. Nonparametric (Kruskal-Wallis) tests, equivalent to ANOVA, are used when the parametric test assumptions are not met. Post-hoc tests comparing multiple groups were performed using Tukey’s test when using ANOVA and Dunn’s test when using Kruskal-Wallis. ns: non-significant, *p < 0.05, **p < 0.01, ***p < 0.001, and ****p < 0.0001

KPC-Luc cells are well established murine PDAC models but are inefficient at metastasizing to the lung and liver. Since PDAC metastasis is the primary cause of mortality in patients, we generated cells to mimic this aspect of PDAC biology which we called metastatic KPC-Ganji & Bassel-Luc (mKPC-GB-Luc) cell line. Figure 1A shows the experimental design for generating this cell line. The human PANC-1 cells exhibit high metastatic potential. mKPC-GB-Luc cell lines were developed from culturing KPC-Luc cells in the filtered conditioned medium of PANC1 cell lines. Authentication using short tandem repeat (STR) analysis confirmed that mKPC-GB-Luc cells were murine cells (data not shown). The mKPC-GB-Luc cell lines showed a high frequency of metastasis after orthotopic implantation. Bulk RNA sequencing revealed that molecular pathways involved in metastasis, including promigration, stemness, mesenchymal, and angiogenic markers, were upregulated in mKPC-GB-Luc cell lines compared to parental KPC-Luc cells (Fig. 1B & Suppl. Figure 1B). Gene ontology (GO) analysis suggests that cell-cell adhesion is downregulated in mKPC-GB-Luc cell lines. (Suppl. Figure 1C). GO molecular function analysis showed that mKPC-GB-Luc cells exhibit higher expression of cytokine receptor binding, suggesting increased inflammatory, autocrine signaling, which are essential for metastasis and tumor progression (Suppl. Figure 1D). These results explain the high metastatic phenotype of mKPC-GB-Luc cells as compared to the parental cell lines. Further assessment of the potential biological relevance of the metastatic phenotype exhibited by mKPC-GB-Luc cell lines was performed by comparing its transcriptomic profile with that of the independently derived metastatic model, KPC-ML1-Luc. Bulk RNA sequencing revealed a high level of transcriptomic correlation between the two independent metastatic models, indicating the presence of common metastasis-related programs in these murine cell lines, irrespective of the induction method used. (Suppl. Figure 1A).

### ProAgio enhances the growth suppression of chemotherapy in the mKPC-GB-Luc orthotopic PDAC models

We assessed the effect of the combination of ProAgio and FOI on tumor growth in the mKPC-GB-Luc orthotopic mouse models (Fig. 1C). The stable body weight of each group of mice suggests that all treatment combinations were well tolerated (Suppl. Figure 2A). Bioluminescent imaging analysis revealed that the combination treatment significantly *(p* < 0.01) decreased the rate of change in bioluminescent signal compared to individual treatments (Fig. 1D). Tumors were isolated (Suppl. Figure 2B) and prepared for downstream analysis. The combination treatment (FOI+ProAgio) resulted in a significant decrease in tumor weight compared to chemotherapy alone, ProAgio alone, or the sham treatment (Fig. 1E).

**Fig. 2:**
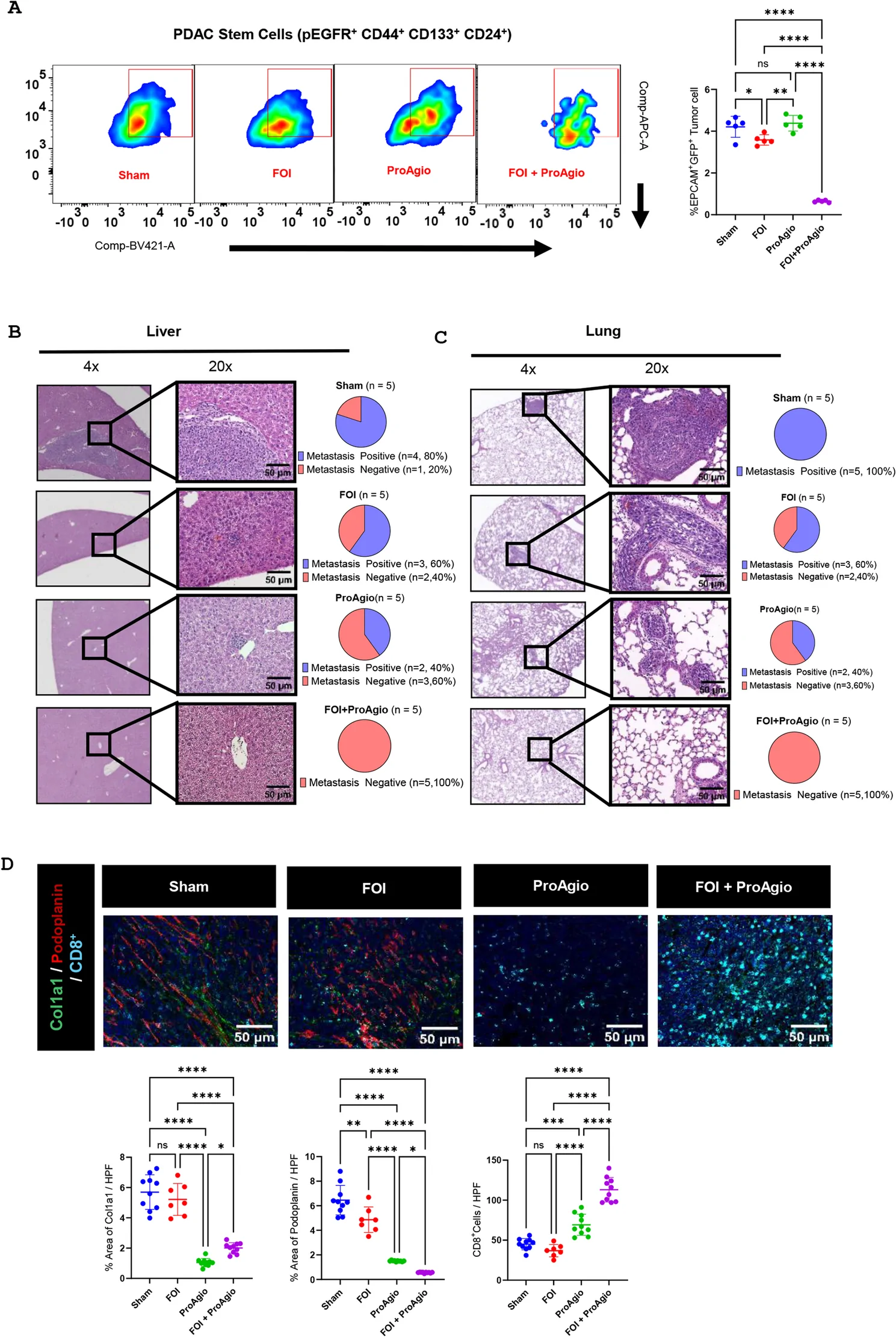
The effects of the combination of chemotherapy plus ProAgio on PDAC metastasis, stemness, and microenvironment in the orthotopic mouse model. **A** mKPC-GB-Luc, tumor stem cells from the primary pancreatic site were identified and quantified using flow cytometry and Flow Jo software. **B** Liver and (**C**) Lung metastasis was observed across different treatment conditions (Sham, FOI, ProAgio, and FOI+ProAgio), and the quantification results are presented in a Phi diagram. Pairwise group comparisons were performed using Fisher’s exact test. To compare trends across ordered groups, the Cochran-Armitage trend test was employed. **D** Representative images of immunohistochemical analysis using fluorescence probes for Col1a1, Podoplanin, and CD8+ across the treatment groups. Scale bars 50 µm. Data presented as mean ± S.D. A one-way ANOVA is used to analyze data across treatment groups when the data are normally distributed. Nonparametric (Kruskal-Wallis) tests, equivalent to ANOVA, are used when the parametric test assumptions are not met. Post-hoc tests comparing multiple groups were performed using Tukey’s test when using ANOVA and Dunn’s test when using Kruskal-Wallis. ns: non-significant, *p < 0.05, **p < 0.01, ***p < 0.001, and ****p < 0.0001

To confirm the effects of ProAgio on hypoxia in the tumor, the pimonidazole hydrochloride study was performed, revealing that ProAgio or a combination of FOI and ProAgio treatment significantly reduced hypoxia (Fig. 1F). This was further confirmed by measuring HIF-1α levels (Fig. 1G). Significant reduction was observed in HIF-1α in ProAgio and FOI+ProAgio compared to FOI or sham treated mice. In mKPC-GB-Luc tumors, ProAgio and ProAgio + FOI significantly increased E-cadherin expression associated with epithelial-type cells, as confirmed by immunofluorescence (Fig. 1G). Given ProAgio’s mechanism of action, integrin β3 was assessed by immunofluorescence and IHC. ProAgio or FOI+ProAgio significantly decreased the expression of integrin β3 compared to sham and FOI-treated mice (Fig. 1G and H). We evaluated mitotic figures by Hematoxylin and Eosin staining and found that FOI+ProAgio treatments showed a significant (*p* < 0.0001) decrease compared with PDAC tumors in FOI-, ProAgio-, or sham-treated mice (Suppl. Figure 2C). To support mitotic decline, we further investigated stemness in mice with PDAC tumors treated with FOI or ProAgio, as well as in sham-treated mice (Fig. 2A). FOI+ProAgio treatment significantly (*p* < 0.0001) decreased stemness compared to FOI or ProAgio-treated animals (Fig. 2A). Overall, our findings support the conclusion that the combination of FOI+ProAgio significantly suppresses hypoxia, inhibits the HIF1α pathway and EMT, and promotes epithelial differentiation in these mKPC-GB-Luc-orthotopic mouse PDAC models.

### Combinational effects of ProAgio plus FOI on metastasis in mKPC-GB-Luc tumor-bearing mice

Metastasis in either the lungs (100%) or the liver (80%) were observed in sham-treated mice (Fig. 2B and C). Metastases were observed in the liver and lung of 3 (*n* = 5; 60%) and 3 (*n* = 5; 60%) animals treated with FOI; respectively. Similarly, metastases were observed in the liver and lung of 2 (*n* = 5; 40%) and 2 (*n* = 5; 40%) of animals treated with ProAgio alone, respectively (Fig. 2B and C). No metastases were observed in the sections from the liver and lung from animals treated with ProAgio plus FOI (Fig. 2B and C). Compared to the combination of sham, there were significantly less metastases observed (0/5 vs. 4/5; Fisher’s Exact test, *p* = 0.0476).

### Combinational effects of ProAgio plus FOI on cancer associated fibroblasts (CAFs) in mKPC-GB-Luc tumors bearing mice

Given the role of CAFs in tumor progression and chemoresistance, we evaluated the effects of the treatments on the CAF subpopulation. Podoplanin is highly expressed in CAFs. Col1a1, is produced mainly by CAFs. ProAgio + FOI treatment significantly (*p* < 0.0001; *p* < 0.05) decreased CAF marker podoplanin and Col1a1 in mKPC-GB-Luc tumor tissue when compared to sham, FOI, and ProAgio-treated mice (Fig. 2D). In order to further characterize the effects of ProAgio on CAF subpopulations, we used flow cytometry. ProAgio + FOI therapy significantly (*p* < 0.0001) increased the percentages of qCAF and antigen-presenting (apCAF), while decreasing the percentages of inflammatory (iCAF) and myofibroblasts (myCAF) (Suppl. Figure 2E). TGFβ activates PSCs and CAFs (Suppl. Figure 2D). In vitro experiments demonstrate that TGFβ stimulated CAF secretions of glycine and serine, and ProAgio inhibited this stimulation. (Suppl. Figure 2D).

### Combinational effects of ProAgio plus chemo regimens on immune cells in mKPC-GB-Luc tumors

The ratio of CD4^+^, CD8^+^, and NK cells, as well as their levels of activation, in the mKPC-GB-Luc microenvironment, was modulated by the combination of ProAgio and FOI. Using IF, we demonstrate an increase in CD8^+^ cells in the FOI+ProAgio-treated group (Fig. 2D). A multiparameter FACS gating strategy for T, NK, ICS, and myeloid panels is shown in supplementary Figs. 3A and 3B. Combination treatment significantly (*p* < 0.0001) increased CD45^+^ cell counts compared with ProAgio, FOI, or sham treatment (Suppl. Figure 4A); no significant difference was observed between the FOI and ProAgio-treated groups. M2 macrophages were significantly (*p* < 0.0001) reduced and M1 polarization (M1/M2 ratio) was significantly (*p* < 0.0001) increased with FOI+ProAgio-treated mice compared to sham and other ProAgio or FOI treated mice (Suppl. Figure 4B). Overall, the myeloid cell population (CD11b cells) significantly (*p* < 0.01; *p* < 0.0001) decreased and increased significantly (*p* < 0.0001) M1 macrophage with combination therapy compared to individual treatments. However, combination treatment significantly increased the overall myeloid population compared with sham, suggesting M1 polarization. Treatment with ProAgio, or the combination, did not affect CD4^+^ Tregs (Suppl. Figure 4C). Treatment with chemo regimen + ProAgio increased the percentages of CD4^+^ and CD8^+^ T cells compared to treatment with chemo or ProAgio alone (Suppl. Figure 4D & 4E). The CD4^+^ and CD8^+^ exhausted T cell subpopulation decreased significantly in combination treatment compared to individual therapies (Suppl. Figure 4 F & 4G). CD4^+^ and CD8^+^ central memory cells significantly increased in combination treatment compared to FOI-treated PDAC cells (Suppl. Figure 4F & 4G). ProAgio, or the combination of FOI and ProAgio treatment, elevated the number of CD8^+^ T cells, as shown by IF analysis (Fig. 2D). A combination of FOI and ProAgio treatment significantly increased the number of TNK cells, but not NK cells, in mKPC-GB-Luc tumors compared to tumors treated with ProAgio or FOI alone (Suppl. Figure 4H). However, NK cells increased in FOI as well as combination therapies compared to sham and ProAgio (Suppl. Figure 4H). T and NK cells were triggered in vitro with PMA and ionomycin in the presence of brefeldin A for 5 h to confirm whether this FOI + ProAgio enhances their functional activity. Cytokine readout was performed using flow cytometry. The results demonstrated intracellular expression of INFγ, CD107α, IL17, and TNFα in CD4^+^, CD8^+^, and TNK cells from animals treated with FOI/ProAgio compared to other groups (results in Suppl Fig. 4K-4 L).

### Combinational effects of ProAgio plus GPTx on tumor growth and metastasis in KPC-ML1-Luc tumor-bearing mice

**Fig. 3:**
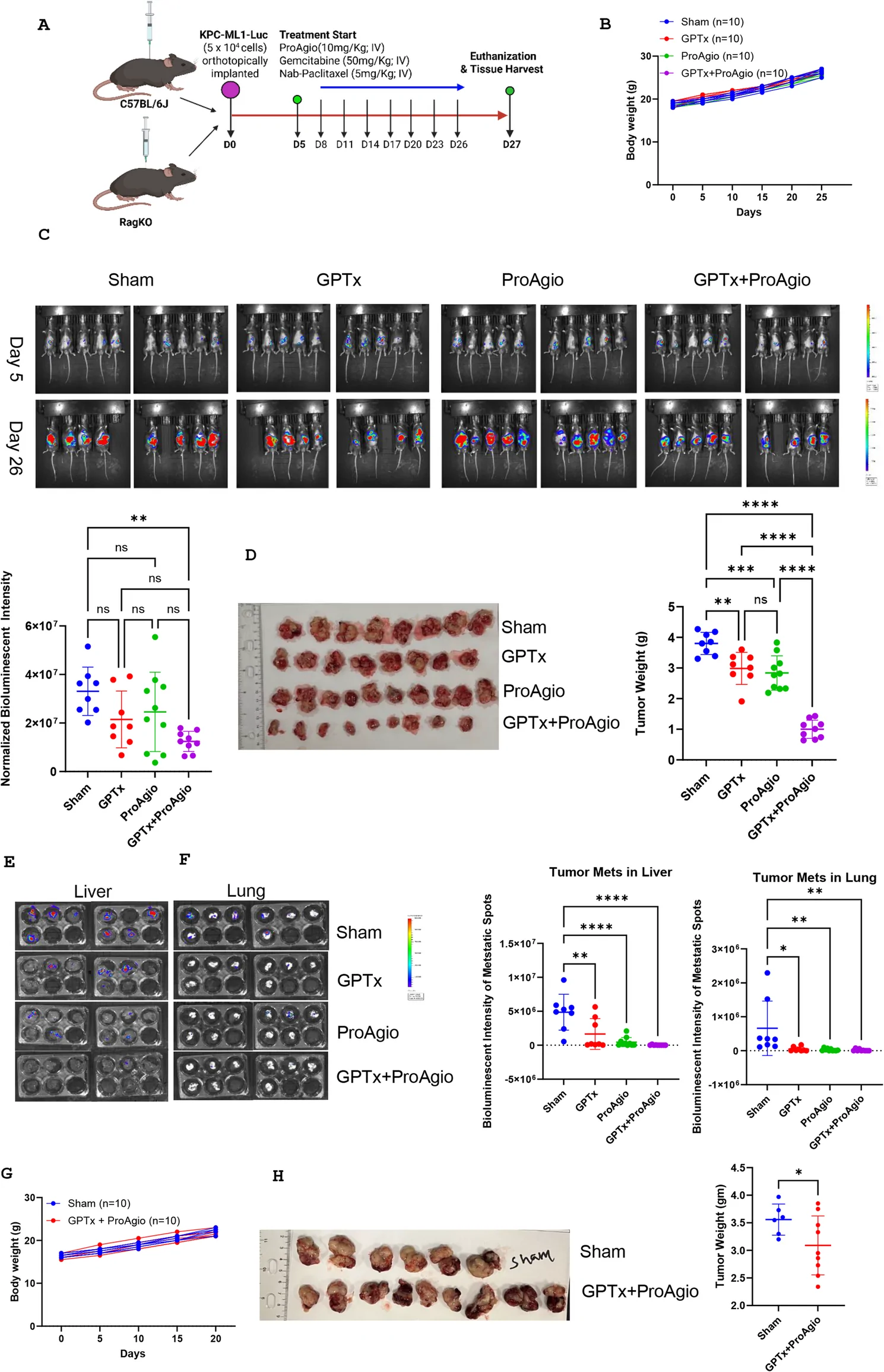
The combination of chemotherapy plus ProAgio on the PDAC metastasis and tumor growth KPC-ML1-Luc orthotopic murine models (wild type and Rag knockout). **A** Schema depicting experimental design of Gemcitabine (G), nab-Paclitaxel (PTx), and ProAgio in the KPC-ML1-Luc orthotopic murine models (Wild type and RagKO). **B** The body weight of each wild-type mouse in the study was measured every 5 days following orthotopic tumor implantation and continuing until study termination. **C** Bioluminescent images of mice with engrafted tumors on Day 5 and Day 26 before euthanizing with quantification of bioluminescent signal as an indicator of tumor growth in vivo (*n*=10 per treatment group). **D** Tumors were shown under various treatment conditions (sham, GPTx, ProAgio, and GPTx+ProAgio). Ex vivo tumor size quantification at termination of the study. **E**, **F** Bioluminescent images of liver and lung metastases after euthanizing sham and treated groups, with quantification of bioluminescent signal as an indicator of metastasis. Data is represented as mean ± SD. A one-way ANOVA test was used to analyze the data across treatment groups, where the data were normal. Nonparametric (Kruskal-Wallis). Tests equivalent to ANOVA were used when the parametric test assumptions were not met. Post-hoc tests comparing multiple groups were performed using Tukey’s test when using ANOVA and Dunn’s test when using Kruskal-Wallis. ns: non-significant, *p < 0.05, **p < 0.01, ***p < 0.001, and ****p < 0.0001. **G** The body weight of each RagKO mouse in the study was measured every 5 days following orthotopic tumor implantation and continuing until study termination. **H** Tumors were shown under various treatment conditions (sham, GPTx+ProAgio). Ex vivo tumor size quantification at termination of the study. Data is represented as mean ± SD. The t-test was used for statistical analysis. *p < 0.05

We tested the effect of gemcitabine (G) plus nab-paclitaxel (PTx) plus ProAgio combination on tumor growth in KPC-ML1-Luc orthotopic mouse models. Schema depicting experimental design of GPTx and ProAgio in the KPC-ML1-Luc orthotopic murine models in C57BL/6J wild type and Rag1-/- knockout (RagKO) mice (Fig. 3A). The stable body weight of each group of mice suggests that all treatment combinations were well tolerated (Fig. 3B). Bioluminescent imaging analysis revealed that the combination treatment significantly *(p* < 0.01) decreased the rate of change in bioluminescent signal compared to sham (Fig. 3C). The combination treatment (GPTx+ProAgio) resulted in a significant (*p* < 0.0001) decrease in tumor weight compared to chemotherapy alone, ProAgio alone, or the sham treatment (Fig. 3D). As expected, liver and lung metastasis were significantly (*p* < 0.0001) decreased in GPTx+ProAgio-treated KPC-ML1-Luc tumor-bearing mice, as confirmed by ex vivo Bioluminescent imaging (Fig. 3E-F).

### Combinational effects of ProAgio plus chemo regimens on immune cells in KPC-ML1-Luc tumors

A multiparameter FACS gating strategy for T, NK, ICS, and myeloid panels is shown in supplementary Figs. 5. Combination treatment significantly (*p* < 0.0001) increased NK, CD4^+^, CD4^+^ effector cells, CD8^+^, δγ T cells, and M1/M2 ratio in ProAgio GPTx treatment compared to control (Suppl. Figure 5). A significant increase in NK, CD4^+^, CD8^+^, and δγ T cells was observed in ProAgio GPTx compared to GPTx. Treatment with ProAgio GPTx decreased T regs compared to GPTx but not compared to control (Suppl. Figure 5D). We did observe an increase in markers of exhaustions (PD1^+^ CTLA4^+^LAG3^+^) in CD4^+^ in ProAgio GPTx compared to GPTx, control, or ProAgio (Suppl Fig. 4D). In CD 8^+^ T cellls there was a significant decrease in PD1^+^ CTLA4^+^LAG3^+^ in ProAgio GPTx compared to GPTx, control, or ProAgio (Suppl. Figure 4 F). Functional stimulation of T and NK cells with PMA and ionomycin in the presence of brefldin A in vivo was performed. An increase in INFγ (in CD4^+^, CD8^+^, γδ T Cell, and NK cells), Granzyme B (in NK, CD8^+^, γδ T Cell), and TNFα (in CD4^+^, CD8^+^, γδ T Cell and TNK cells) was observed in the immune cells from animals treated with GPTx/ProAgio compared to other groups (Suppl Fig. 5G-5I).

### Rag 1 -/- KO study

The flow cytometry data from both orthotopic models suggested immune-mediated anti-tumor activity, with an elevated frequency of functional cytotoxic T lymphocytes (CTLs). To assess the contribution of the CTLs to the observed anti-tumor effects of ProAgio+GPTx, we used Rag1-/- KO mice. At the end of the experiment, only 6 of the 10 animals in the sham-treated group survived, whereas 9 of the 10 in the ProAgio+GPTx-treated group survived. The 4 animals in the sham died due to high tumor burden. The stable body weight of each group of mice suggests that all treatment combinations were well tolerated (Fig. 3G). Although ProAgio+GPTx significantly (*p* < 0.05) decreased the tumor weight compared to sham treatment in RagKO mice (Fig. 3H), the magnitude of the effect of ProAgio+GPTx was larger in the wild-type immune competent mice (Fig. 3D). These results suggest that the observed antitumor activity of ProAgio+GPTx is largely in part mediated by tumor-infiltrating CTLs in wild-type immune competent mice.

### Effect of gemcitabine (Gem) combinations with ProAgio on PDAC growth and survival in KPC GEMMs

**Fig. 4:**
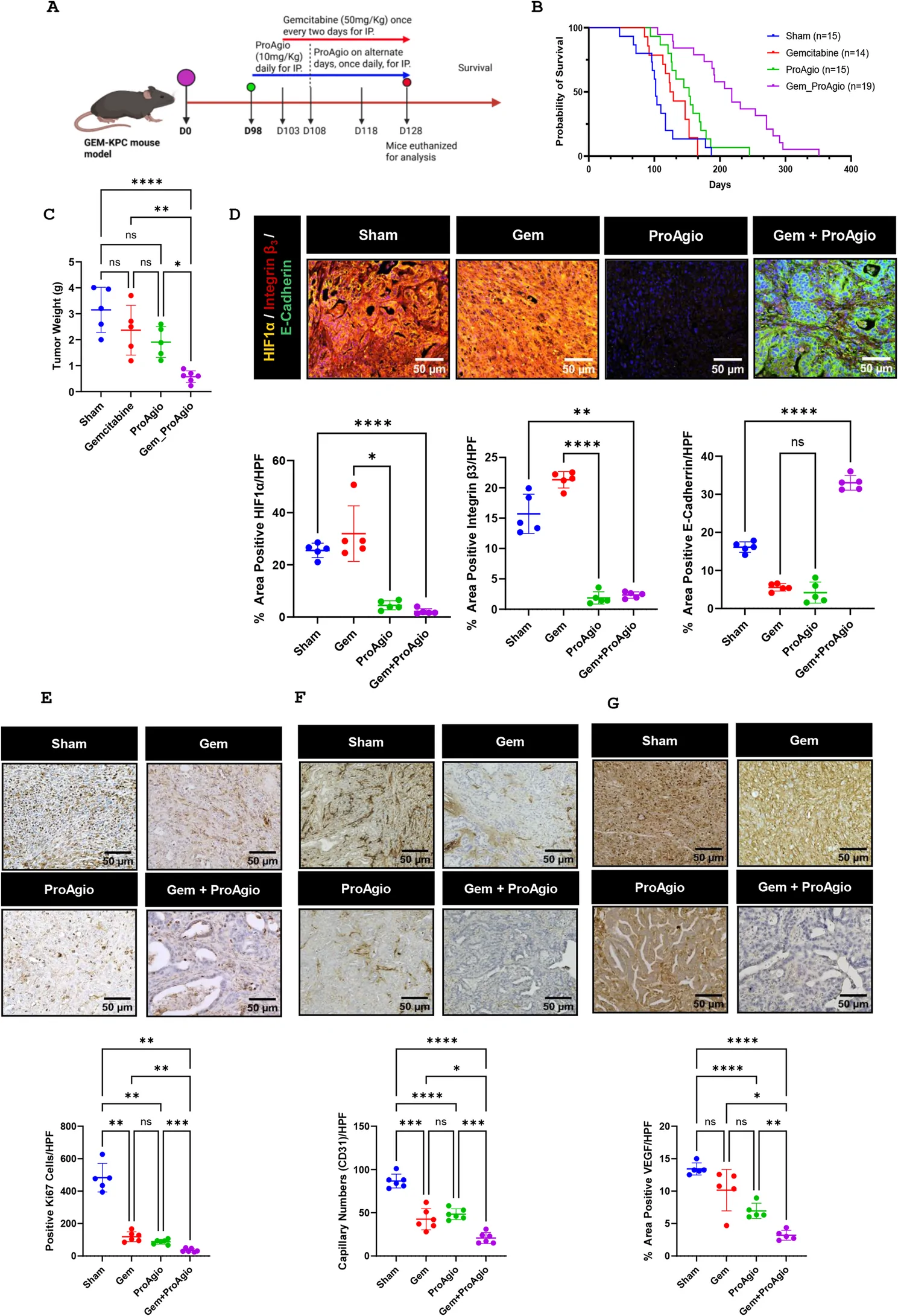
The effects of the combination of chemotherapy plus ProAgio on PDAC progression in the genetically engineered mouse model (GEMM). **A** Schema depicting experimental design of gemcitabine and ProAgio in the GEMM. **B** Kaplan-Meier survival curves for the GEMM. Gemcitabine plus ProAgio treatment significantly (*p* < 0.0001) increased survival in mice compared with sham, individual Gem, or ProAgio treatments. **C** Quantification of tumor weights across treatment groups in the GEMM used for single-cell RNA sequence analysis. **D** Representative images of immunohistochemical analysis using fluorescence probes for HIF1α, integrinβ3, and E-cadherin across the treatment groups. **E**-**G** Representative Immunohistochemistry images of Ki67, capillary number (CD31), and VEGF, along with quantification across treatment groups. Scale bars 50 µm. Data is represented as mean ± SD. A one-way ANOVA test was used to analyze the data across treatment groups, where the data were normal. Nonparametric (Kruskal-Wallis). tests equivalent to ANOVA were used when the parametric test assumptions were not met. Post-hoc tests comparing multiple groups were performed using Tukey’s test when using ANOVA and Dunn’s test when using Kruskal-Wallis. ns: non-significant, *p < 0.05, **p < 0.01, ***p < 0.001, and ****p < 0.0001

In this study, we employed two sets of experiments in GEM-PDAC models: the first for survival analysis (Fig. 4A) and the second for mechanistic evaluation. The combination treatment significantly (*p* < 0.0001) increased (~ 350 days) survival, decreased terminal tumor weight and liver metastatic spots in GEM-PDAC models (Fig. 4B; Suppl. Figure 6 A-B) compared to vehicle, gemcitabine or ProAgio treated groups.

Next, we assessed the efficacy of gemcitabine and ProAgio on PDAC progression in GEM-PDAC models. Gem+ProAgio significantly (*p* < 0.0001) reduced tumor weight (Fig. 4C). Individual treatments (Gem or ProAgio) reduced the average tumor weight; however, there was no significant difference in tumor weight between individual treatments compared to sham (Fig. 4C). These tumors were used for further downstream scRNA-seq, IHC, and IF analysis. IF analysis showed that Gem+ProAgio significantly decreased HIF1α and integrin β3, and significantly (*p* < 0.0001) increased E-cadherin, compared to the sham and Gem group (Fig. 4D). Furthermore, ProAgio significantly decreased HIF-1α, integrin β3, and E-cadherin compared to the sham group. IHC analysis revealed that Ki-67 positive cells, capillary number (CD31 positive), and VEGF expression were significantly lowered in Gem+ProAgio treated mice (Fig. 4E-G) compared to other individual treatments.

### The combination of Gem+ProAgio inhibits the PDAC cell progression and metastasis

**Fig. 5:**
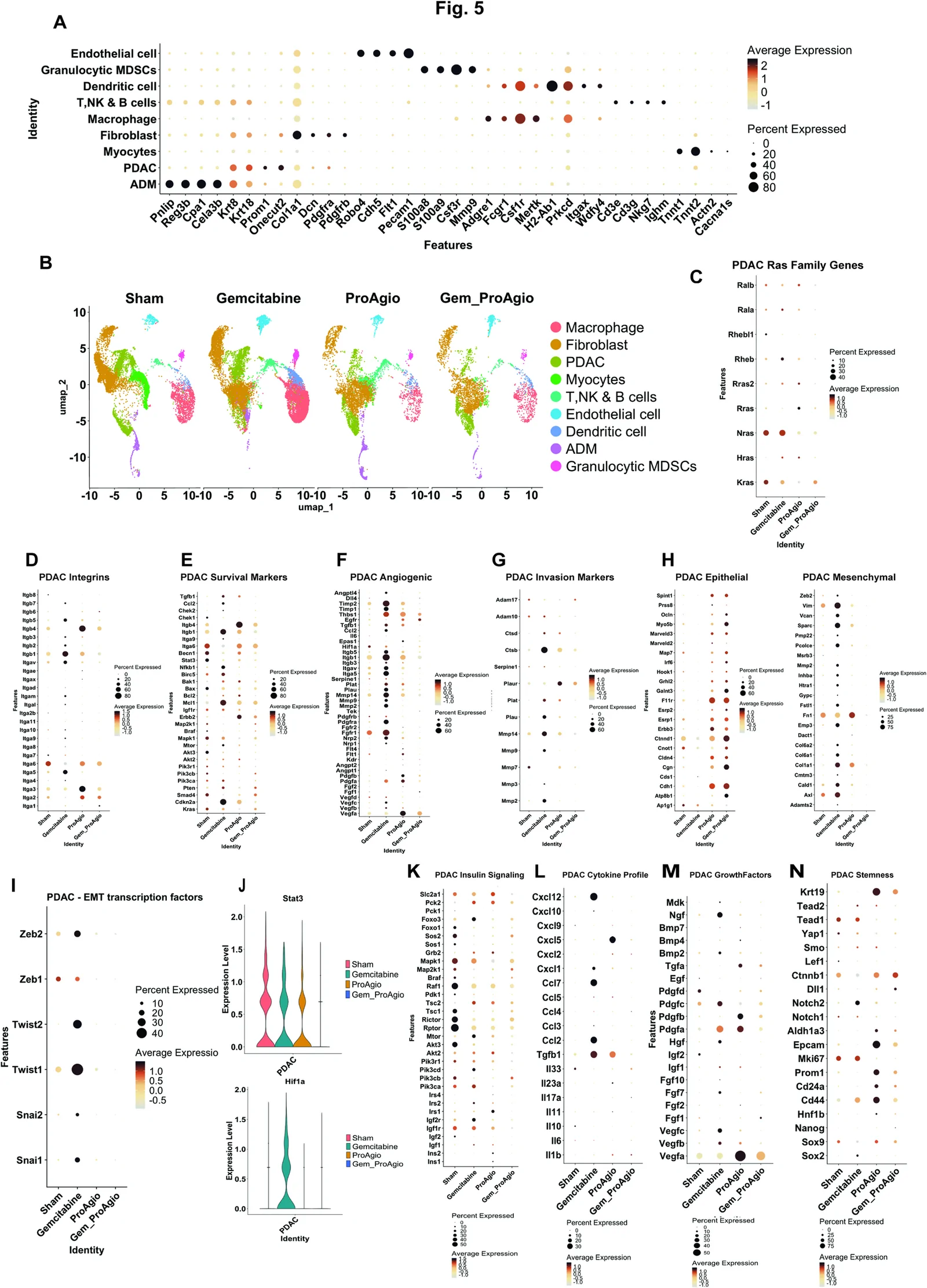
Single-cell RNA sequencing analysis reveals a marked reduction in GEM-PDAC-model treated with Gem, ProAgio, and Gem+ProAgio. **A** Dot plots exhibit well-known 36 gene markers, indicating nine main cell types. **B** The UMAP analysis shows that Gem+ProAgio treatment in the GEM-PDAC model decreased all clusters. **C**-**G** The dot plot shows Gem+ProAgio treatment decreases the RAS family, integrins, survival, angiogenic, and invasion gene markers compared to all other treatments. **H**, **I** The dot plot showed that the Gem+ProAgio treatment increased epithelial and decreased mesenchymal, as well as EMT, transcriptional markers compared with all other treatments. **J** The violin shows transcription factors STAT3 and HIF1α. **K** The dot plot showed that Gem+ProAgio treatment decreased insulin signaling markers. **L**-**N** The dot plot showed that Gem+ProAgio treatment decreased the expression of cytokine and growth factor gene markers as well as stemness markers, compared to all other treatments

To better understand the reduction in PDAC growth, single-cell RNA sequencing (scRNA-seq) was performed. After quality control, using normalized scRNA-seq, Uniform Manifold Approximation and Projection (UMAP) analysis revealed cellular heterogeneity and identified 0–17 clusters using the Louvain clustering algorithm. The clusters were clearly separated and identified as nine cell types using well-established mouse gene markers reported in the literature (Fig. 5A). The labelled cell types were granulocytic MDSCs, macrophages, dendritic cells, myocytes, endothelial cells, TNK (T cells, natural killer (NK) cells, B cells) cells, fibroblasts, ADM, and PDAC (Fig. 5A). UMAP analysis revealed that Gem+ProAgio treatment decreased the proportion and number of PDAC cells, as well as other cell types, compared with sham and individual treatments (Gem or ProAgio) (Fig. 5B; Suppl. Figure 6 C). These observations are consistent with the H&E stain results (Suppl. Figure 2 C). IHC results revealed that Gem+ProAgio treatment significantly (*p* < 0.0001) reduced proliferation and angiogenesis as measured by Ki-67, CD31, and VEGF, compared with other individual therapies (Fig. 4E-G).

Gem+ProAgio treatment significantly (*p* < 0.05) decreased the RAS family markers, integrin pathway, migration and adhesion markers compared to other individual treatments (Gem, ProAgio, or sham) in PDAC clusters (Fig. 5C-D; Suppl. Figure 6D). Interestingly, we found that Gem significantly (*p* < 0.05) induced markers of the RAS and integrin pathways in PDAC clusters. These observations suggest the RAS/Integrin/HIF1α/STAT3 axis may be a survival pathway for Gem treatment in PDAC (Fig. 5E). Dot plot analysis revealed that integrin, ER stress, and autophagy pathway gene signatures decreased significantly (*p* < 0.05) in the combination treatment (Gem+ProAgio) compared with individual treatments in PDAC, ADM and endothelial clusters (Fig. 5D; Suppl. Figure 6E & 6 F, 7 A-7 C, 8 A-8 C). Next, we evaluate survival and other markers associated with metastasis in PDAC, ADM, and endothelial clusters. Dot plot analysis revealed that Gem+ProAgio significantly (*p* < 0.05) reduced the survival markers in both PDAC and ADM clusters compared to G, ProAgio, or sham (Fig. 5E; Suppl. 7D & 8D). Gem+ProAgio also decreased the expression of angiogenesis, invasion, and migration markers in both PDAC and ADM clusters, compared with other individual treatments (Fig. 5F and G; Suppl. Figure 6D, 7E, 7 F, 7G, & 8E).

EMT is essential for PDAC progression [17]. Gem+ProAgio therapy increases epithelial markers and decreases mesenchymal markers compared with other individual treatments (Fig. 5H). High expression of mesenchymal markers was observed in Gem-treated PDAC and ADM clusters (Fig. 5H; Suppl. Figure 7 H). In addition, EMT is regulated by transcriptional factors (Zeb1/2, Snail1/2, and Twist1/2), which are primarily induced under Gem conditions. Gem+ProAgio significantly reduced the expression of EMT transcription factors compared to other treatment conditions in PDAC as well as in ADM (Fig. 5I; Suppl. Figure 7 H). This data was further supported by increased E-cadherin expression in combination-treated PDAC tissues. Immunofluorescence analysis supports the scRNA-seq cells data. E-cadherin was upregulated in combination-treated PDAC tissues (Fig. 4E).

Transcription factors, cytokines, and growth factors play a key role in highly regulating angiogenesis [18]. Angiogenesis markers were elevated in Gem-treated PDAC compared to other individual treatments (Fig. 5F; Suppl. Figure 7 F & 8E). Gem+ProAgio significantly reduced the angiogenic markers compared to other treatment conditions in PDAC clusters. HIF-1α and STAT3 are transcription factors that act as master regulators of angiogenesis. They were also downregulated in the combination treatment (Gem+ProAgio) and highly upregulated in the sham group for PDAC clusters (Fig. 5J). Insulin, Cytokine, and growth factor gene signatures were also downregulated in Gem+ProAgio-treated PDAC, ADM, and endothelial (cytokine and growth factors only) clusters (Fig. 5K, L and M; Suppl. Figure 7I, 7 J, 7 K, 8 F & 8G). Similar results were also observed in animal tumors and serum samples (Suppl. Figure 9).

Stemness and Ras/Wnt/insulin signaling contribute to PDAC and ADM progression, survival, and resistance (Fig. 5C, K and N; Suppl. Figure 7I and 7 L). Combination (Gem+ProAgio) therapy significantly reduced all these pathways and stemness gene signatures in both PDAC and ADM clusters (Fig. 5C, K and N; Suppl. Figure 7I and 7 L). These observations suggest that the ProAgio combination with chemotherapy potentially inhibits stemness (Fig. 2A).

### The combination of Gem+ProAgio reprograms the cancer-associated fibroblasts (CAFs)

**Fig. 6:**
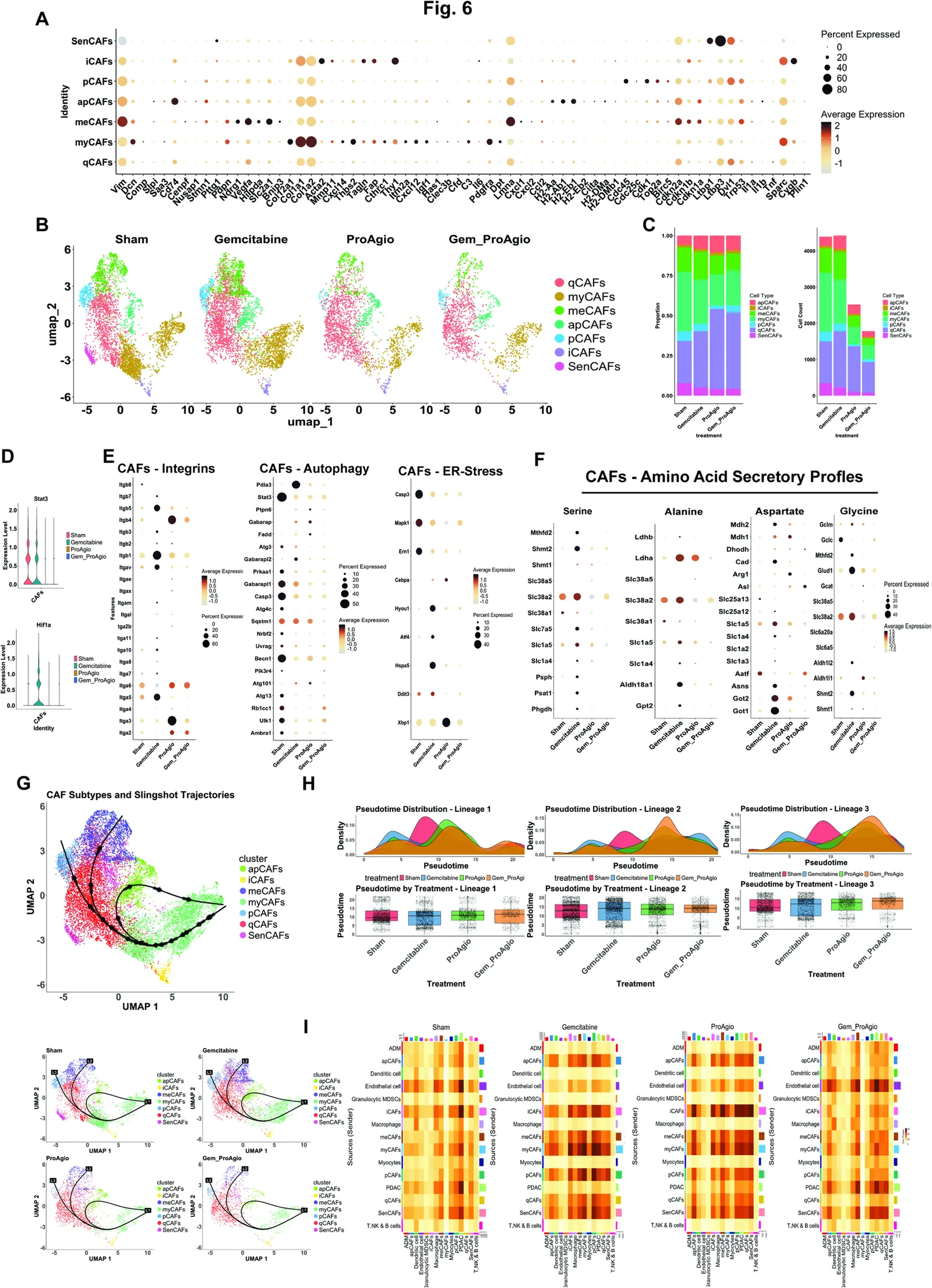
Gemcitabine (G) with ProAgio reduces the activated CAF phenotype and increases qCAFs in the GEM-PDAC models. **A** Dot plots exhibit well-known 72 gene markers, indicating seven main cell types. **B**, **C** The UMAP analysis showed that ProAgio or Gem+ProAgio treatment in the GEM-PDAC model increases qCAFs and decreases all other CAF subtypes. Cell counts and proportions of CAF subsets as determined by single-cell RNA sequencing. **D** The violin plot shows that ProAgio or Gem+ProAgio treatment decreased the expression of STAT3 and HIF1α gene markers compared to all other treatments. **E** The dot plot shows that Gem+ProAgio treatment decreased the expression of integrin, autophagy, and ER stress gene markers compared to all other treatments. **F** The dot plot shows that the Gem+ProAgio treatment decreases the expression of secretory gene markers for CAFs, such as Serine, Alanine, Aspartate, and Glycine pathway-associated genes, compared to all other treatments. **G**, **H** Slingshot Trajectories and Pseudotime distribution analysis show that active CAFs originate from the qCAFs pool, differentiate into functionally distinct subtypes, and are found in various regions of the trajectory. In Gem drives, CAFs are directed toward senCAFs (terminal state), whereas ProAgio alone or Gem+ProAgio appears to reprogram them toward qCAFs or to restrain that trajectory. ProAgio alters lineage flow and reduces iCAFs and myCAFs. Furthermore, Gem+ProAgio redirects or collapses the lineage. **I** Cell-cell interaction analysis indicates that Gem+ProAgio reduced interactions among TME components

Next, we subsetted the fibroblast clusters and classified them into seven types of subclusters [quiescent CAFs, qCAFs, myofibroblast-like CAFs (myCAFs), metabolic CAFs (meCAFs), antigen-presenting CAFs (apCAFs), proliferative CAFs (pCAFs), inflammatory CAFs (iCAFs), and senescent CAFs (senCAFs)] using well-characterized markers (Fig. 6A) [19–21]. UMAP, cell count, and proportion analysis suggest that Gem+ProAgio treatment induces CAFs to reprogram into qCAFs (Fig. 6B-C). Two major transcriptional factors, HIF1α and STAT3, showed reciprocal activation in PDAC-TME. For instance, HIF1α increases IL6, which in turn activates STAT3. Activated STAT3 can increase HIF1α and its stability in CAFs and tumor cells [22, 23]. Both are important for invasion, angiogenesis, and immune suppression. Violin plot analysis revealed that the Gem+ProAgio treatment significantly reduced STAT3 and HIF1α expression compared with the other individual treatments (Fig. 6D). These scRNA-seq data closely align with our FLOW data (Fig. 3B). Dot plot analysis revealed that integrin, autophagy, and ER stress pathway gene signatures decreased significantly (*p* < 0.05) in the combination treatment (Gem+ProAgio) compared to individual treatments in both PDAC and endothelial cells (Fig. 6E; Suppl. 7 A – 7 C; Suppl. 8 A – 8 C).

CAFs remodel the PDAC-TME and influence PDAC survival, progression, and resistance to therapy. CAFs secrete serine, glycine, alanine, and aspartate, which are essential for DNA synthesis and replication, the TCA cycle for ATP production, and collagen synthesis, thereby contributing to PDAC proliferation and resistance to therapy [24, 25]. Gem+ProAgio treatment significantly reduces all four pathway gene signatures (Fig. 6F). These results were validated in vitro for serine and glycine. ProAgio significantly decreased the serine and glycine in activated PSCs (Suppl. Figure 2D).

Pseudotime analysis of CAF address heterogeneity, differentiation trajectories, and functional states within the PDAC-TME. UMAP projection shows CAFs under four treatment conditions (sham, ProAgio, Gem, and Gem+ProAgio). We found that qCAFs are in the trajectory beginning area. senCAFs and meCAFs are situated in the top part of the trajectory and are connected to pCAFs, and qCAFs. myCAFs are located on the right negative side of the trajectory, and iCAFs are located right down the side and connected to myCAFs (Fig. 6G). These observations revealed that active CAFs originate from the qCAFs pool, differentiate into functionally distinct subtypes, and are found in various regions of the trajectory. Gem treatment drives CAFs towards senCAFs (terminal state), whereas ProAgio alone or Gem+ProAgio appears to reprogram them to qCAFs or to restrain that trajectory. ProAgio alters lineage flow and reduces iCAFs and myCAFs. Furthermore, Gem+ProAgio redirects or collapses the lineage (Fig. 6G and H**)**. It also elevates the dynamic plasticity that could allow CAFs to adapt to PDAC metabolic pressures. Combination treatment reprograms active CAFs to inactive CAFs (qCAFs), as confirmed by scRNA-seq and flow cytometry analysis (Fig. 3B). Cell-cell communication analysis suggested that Gem+ProAgio treatment decreases the interaction between CAFs and PDAC compared to Gem-treated mice (Fig. 6I).

### The combination of Gem+ProAgio induces M1 polarization

**Fig. 7:**
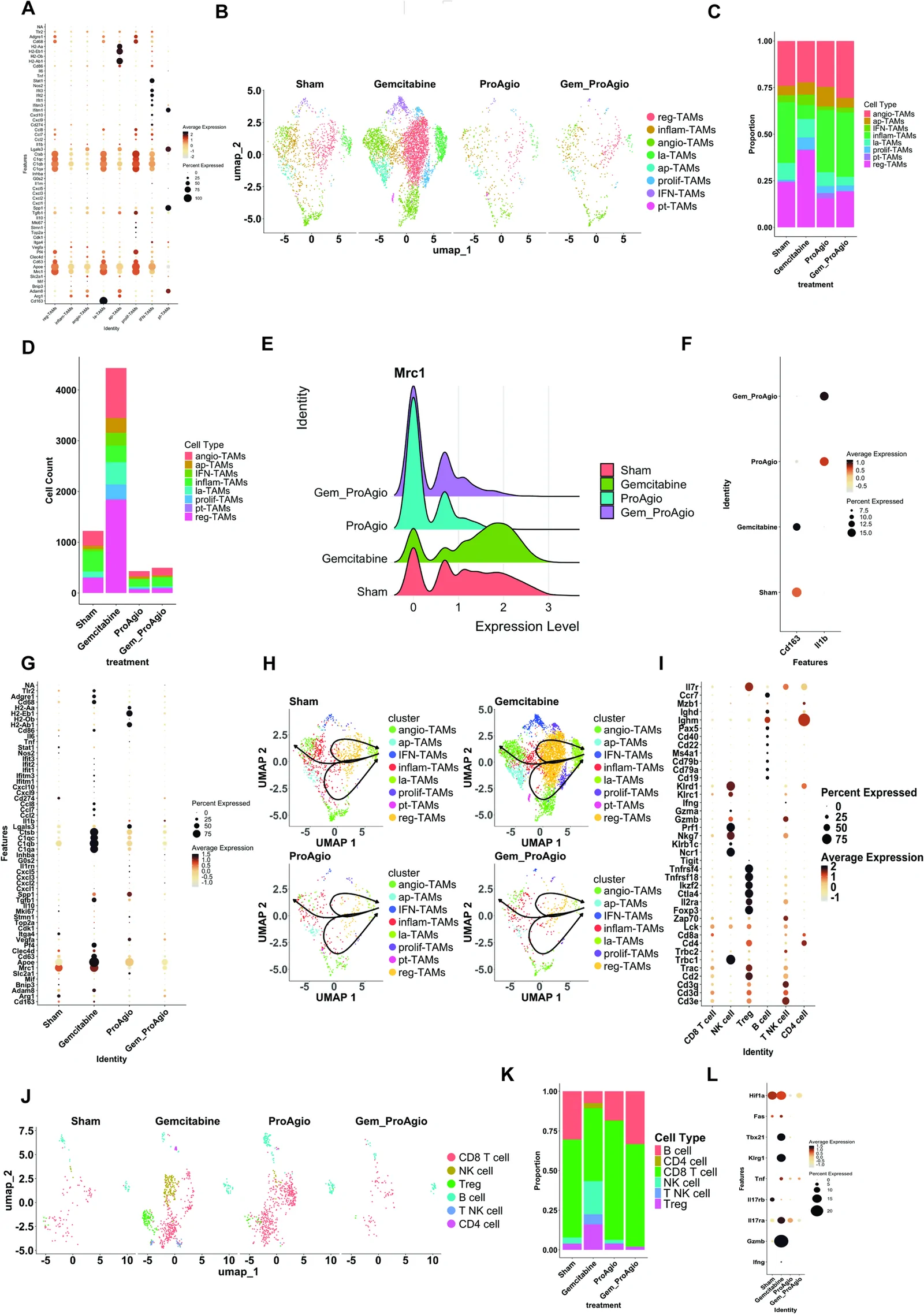
Gemcitabine (G) with ProAgio reduces the tumor-associated macrophages (TAMs) and increases the infiltration of immune cells in the GEM-PDAC models. **A** Dot plots show well-known gene markers associated with various types of TAMs. **B**-**D** The UMAP, cell count, and proportional analysis show that ProAgio or Gem+ProAgio treatment in the GEM-PDAC model decreases all TAM subtypes. Gem increases regulatory TAMs. **E** ProAgio or Gem+ProAgio treatment in the GEM-PDAC model decreases the Mrc1 (M2 macrophage) gene marker compared with Gem and sham. **F**, **G** The dot plots show that ProAgio or Gem+ProAgio treatment decreases CD163 and TAM-associated gene markers and increases proinflammatory cytokine IL-1β and M1 gene markers compared to all other treatments. **H** Slingshot Trajectories and Pseudotime distribution analysis show that ProAgio alone or Gem+ProAgio appears to reprogram them to proinflammatory TAMs (M1) or restrain that trajectory. ProAgio alters lineage flow and reduces TAMs. Furthermore, Gem+ProAgio redirects or collapses the lineage. **I** Dot plots exhibit well-known gene markers associated with various types of T and NK cells. **J**, **K** The UMAP and proportional analysis show that ProAgio or Gem+ProAgio treatment in the GEM-PDAC model decreased Tregs and increased B and CD8+ cells. Gem increased regulatory T cells and T NK cells. **L** The dot plots showed that the ProAgio or Gem+ProAgio treatments decreased effector gene markers compared to all other treatments. Gem significantly (*p*<0.05) increases effector transcript levels compared to other treatment conditions

We subclustered tumor-associated macrophages (TAMs) using well-established markers and identified eight distinct TAM subtypes: angioTAMs, apTAMs, laTAMs, ifnTAMs, inflamTAMs, prolifTAMs, ptTAMs, and regTAMs (Fig. 7A). The total number of TAMs decreased significantly in both ProAgio with and without Gem (Fig. 7B). The apTAMs, ifnTAMs, prolifTAMs, ptTAMs, and regTAMs were highly increased in the tumors of Gem-treated mice. In the proportional and cell count analyses, the graph showed that regTAMs significantly decreased in ProAgio- and ProAgio + Gem-treated tumors compared with sham- and Gem-treated PDAC tumors (Fig. 7C and D). Violin and Dot plot analyses revealed that the M2-associated gene signature was highly expressed in Gem-treated PDAC mice compared with other treatments (Fig. 7E -G). M1-associated gene signatures are highly expressed in ProAgio and Gem+ProAgio (Fig. 7F). Our flow data also suggested that chemo+ProAgio induced M1 polarization (Suppl. Figure 4B).

Slingshot analysis revealed that in the sham, the trajectory initiates ap and angio TAMs before branching off. One lineage proceeds from reg-TAMs to Prolif-TAMs, whereas another goes through IFN- and inflame-TAMs (Fig. 7H). This implies that TAMs are adaptable, branching into immunosuppressive and inflammatory/IFN-driven pathways. The G-treated group’s trajectories were comparable to those of the sham group, but with a larger emphasis on the reg-TAMs/Prolif-TAMs lineage (Fig. 7H). These findings indicate that Gem administration biases TAM differentiation toward immune-suppressive states. In ProAgio-treated groups, the trajectories appeared trimmed, and the immunosuppressive lineage (reg-TAMs/Prolif-TAMs) was either weak or absent (Fig. 7H). The remaining cells are primarily associated with the inflammatory/IFN-TAM axis. This suggests that ProAgio alters the fate of TAMs, preventing them from developing into the most protumor lineages. In the Gem + ProAgio-treated group, the density was midway between the Gem and ProAgio groups, showing limited flexibility and a move away from immunosuppressive differentiation.

### The combination of Gem+ProAgio activates CD8^+^ T cells

Next, we subsetted T and B cells and classified T cells into seven types using well-established markers reported in the literature. They are CD8^+^, CD4^+^, NK cells, B cells, TNK cells, and Tregs (Fig. 7I). scRNA-seq analysis revealed that ProAgio or Gem+ProAgio treatment significantly (*p* < 0.05) increased the expression of CD8 + cells compared with FOI treatments and sham (Fig. 7J and K). Furthermore, we found that Tregs decreased significantly (*p* < 0.05) in PDAC tumors from GEM-PDAC mice treated with Gem+ProAgio (Fig. 7K). Interestingly, FOI+ProAgio treatment significantly increased the expression of CD4^+^, CD8^+^, and T NK cells compared with individual therapy in the mKPC-GB-Luc orthotopic mouse model (Suppl. Figure 4D, 4E, & 4 H). Gem treatment increased the expression of effector gene markers Fas, Tbx21, Klrg1, Tnf, IL17ra, Gzmb, and Ifng, and decreased the expression of IL17rb and HIF-α (Fig. 7L). Interestingly, ProAgio and ProAgio + Gem significantly reduced all gene signatures, primarily by reducing tumor size. Low HIF expressions may indicate higher levels of CD8^+^ cells in PDAC-TME.

### Sequential multiplex immunofluorescence (COMET) reveals Gem+ProAgio-mediated immune activation and CAF remodeling in the KPC GEM model

**Fig. 8:**
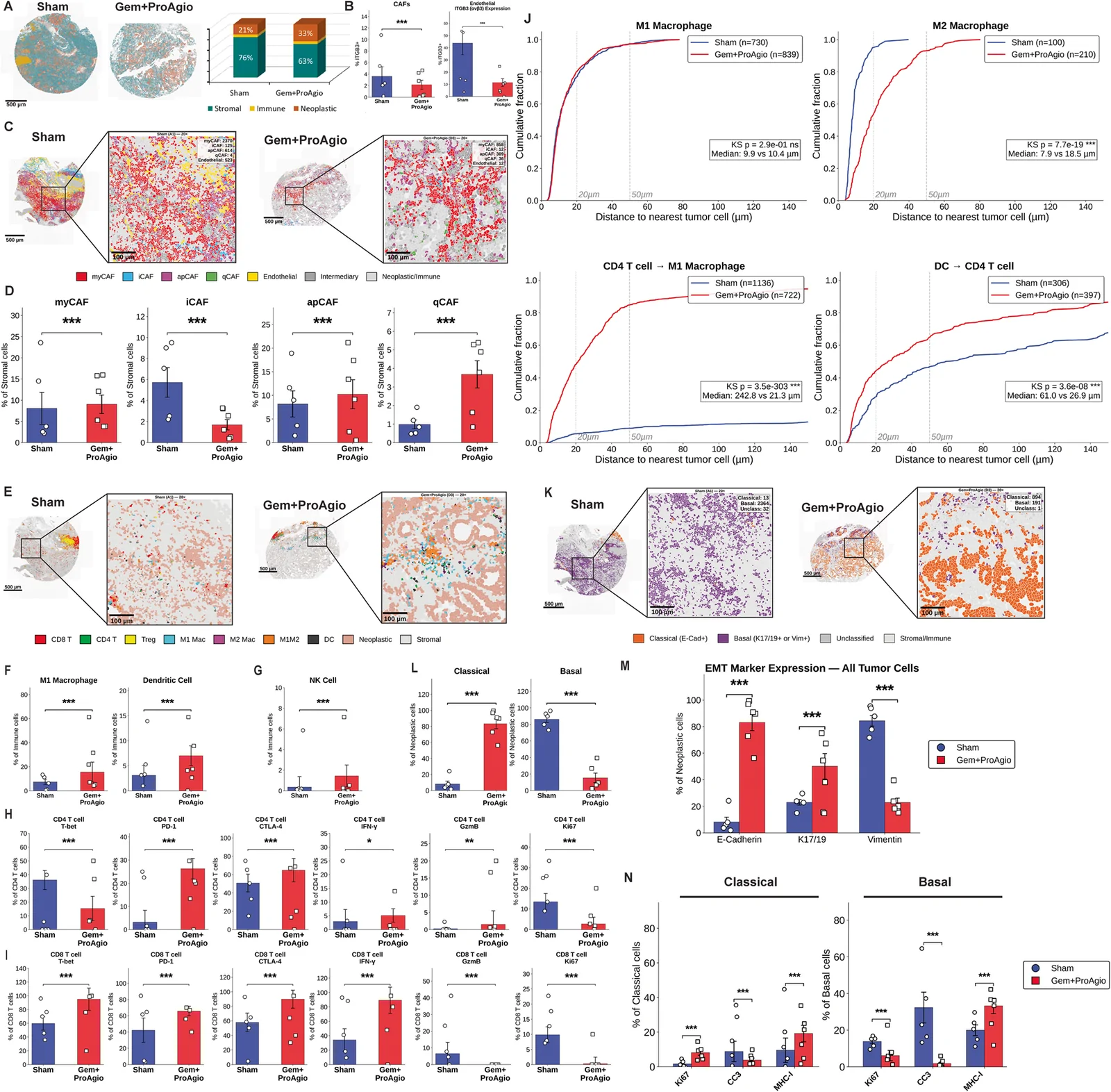
Spatial remodeling of the TME (tumor microenvironment) following Gem+ProAgio treatment in the KPC GEM model. **A** Spatial maps show the distribution of neoplastic cells, immune cells, and stromal cells in sham and Gem+ProAgio-treated tumors. Proportion of stromal, immune, and neoplastic components in both sham (*n*=5) and Gem+ProAgio (*n*=6) treated groups. **B** Integrin β3 expression in cancer-associated fibroblasts (CAFs), endothelial cells **C**, **D** Subpopulation of CAFs and quantification of CAFs. **E** Immune compartments and their functional state. **F** Myloid cells' functional state. **G** NK cell functional state. **H** CD4+ T cells' functional state. **I** CD8+ T cells' functional state. **J** Spatial proximity assessment of tumor–M1 and M2 macrophages and CD4+ T cell–M1 macrophage and DC to CD4+ T cells. **K** Spatial maps showing the distribution of cancer cells, such as Classical type (expressing E-cadherin and Basal type (expressing K17/K19 or vimentin) in sham and Gem+ProAgio treated tumors. **L** Quantification of cancer cells in both sham (*n*=5) and Gem+ProAgio (*n*=6) treated groups. **M** Quantification of EMT markers E-cadherin, vimentin, and K17/K19. **N** Quantification of Ki67 and MHC1 in both classical and basal types in the sham and Gem+ProAgio-treated tumors

The COMET study revealed distinct cellular neighborhoods and communication networks in sham versus Gem+ProAgio-treated GEM-PDAC tumors. Gem+ProAgio-treated tumors showed a transformed spatial manner. Quantitative assessment showed a reduction in proportion of stromal cells (CAFs and CD31^+^ endothelial cells) in Gem+ProAgio-treated tumors compared with sham-treated tumors (Fig. 8A). The expression of integrinβ3 in CAFs and endothelial cells significantly decreased in Gem+ProAgio-treated tumors compared to sham (Fig. 8B). The subpopulation of CAFs, qCAFs, significantly increased, and iCAFs significantly decreased in Gem+ProAgio-treated tumors (Fig. 8C-D). In the immune compartment, Gem+ProAgio treatment increased M1 polarization, dendritic cells, NK, CD4^+^, CD8^+^, and exhausted (PD-1, CTLA-4) markers (Fig. 8E-I).

Spatial proximity investigation supported increased interactions between CD4^+^ T cells and residual cancer cells, indicating improved immune activation (Fig. 8J). DCs are very close to CD4^+^ T cells and M1 macrophages within a 20 μm radius, indicating enhanced antigen presentation and priming in Gem+ProAgio-treated tumors (Fig. 8J). Importantly, the distribution of M2 macrophages shifted away from tumor cores in Gem+ProAgio-treated tumors. Enriched M1 macrophages also reside within a 20 μm radius of the tumor cells in Gem+ProAgio-treated tumors (Fig. 8J), suggesting increased immune activation. There is an increase in MHC-1 expression (Fig. 8N) which could be involved in inducing antitumor immunity.

In the sham tumors, the basal (highly vimentin-expressing) tumor cells are high, whereas the epithelial marker E-cadherin (highly expressed in Classical type) are low (Fig. 8K-M). Ki67 is also highly expressed in basal-type tumor cells (Fig. 8N). Gem+ProAgio treatment inhibited EMT and induced E-cadherin expression (Fig. 8M-N).

### The combination of Gem+ProAgio modulates cytokines and growth factors in serum and tumors in GEM-PDAC models

ELISA results revealed that the combination of Gem plus ProAgio treatment significantly decreased VEGF, IGF1, CXCL2, GM-CSF, G-CSF, TGFβ, and EGF in both serum and tumor samples in GEM-PDAC models compared to Gem- or sham-treated GEM-PDAC models (Suppl. Figure 9). These results are consistent with single-cell RNA sequence data (Fig. 5L and M).

## Discussion

In the current study, ProAgio in combination with standard of care chemotherapy regimens, induced CAF reprogramming, modulated angiogenesis, improved hypoxia, and inhibited PDAC growth and metastasis. More importantly, ProAgio plus chemotherapy significantly extended survival in KPC GEM model compared with either therapy alone.

Our results support previously published literature indicating that conventional chemotherapies do not modulate key factors that impact resistance, such as hypoxia, the immune microenvironment, and EMT [26]. Importantly, EMT transcription factors, including Snail, Slug, and ZEB1, can be suppressed by oxidative stress and cellular energy depletion under prolonged hypoxic conditions [27]. Furthermore, oxidative stress and DNA damage brought on by chemotherapy can trigger p53 signaling, which maintains epithelial properties and prevents EMT [28]. Chemotherapy-triggered hypoxia arises from microenvironmental and vascular remodeling that occurs due to cytotoxic chemotherapy and can contribute to the emergence of resistance [29]. Besides, chemotherapy can elevate fibrosis in the tumor, consequently increasing interstitial fluid pressures, which together contribute to a hypoxic TME [30]. As an adaptive mechanism to oxygen deficiency, post-chemotherapy pancreatic tumors frequently demonstrate elevated levels of HIF-1α [31, 32]. Thus, the ensuing hypoxic TME stabilizes HIF-1α in both tumor and stromal cells, initiating transcriptional programs that support survival, angiogenic signaling, and metabolic shift. These observations help explain why the effects of conventional chemotherapy are transient, with the inevitable emergence of resistance and metastasis, and highlight the importance of developing novel agents that can modulate the TME. In this study, we clearly demonstrate that ProAgio can alter these effects by simultaneously targeting activated CAFs and endothelial cells, thereby promoting less hypoxia in the TME. This, in turn, leads to inhibition of HIF-1α, EMT, and other mechanisms contributing to the emergence of resistance to chemotherapy.

Desmoplasia in PDAC is mediated by CAFs [33]. CAFs undergo phenotypic reprogramming under hypoxic stress, characterized by increased production of cytokines, such as IL-6 and CXCL12, profibrotic factors, including TGF-β and CTGF, and ECM proteins, such as collagen I and fibronectin [34]. Chemotherapy and hypoxia work together to increase CAF activation [22, 23]. For instance, through HIF-1α- and TGF-β-dependent signaling, hypoxia activates CAFs to assume a more contractile, myofibroblastic nature. Additionally, chemotherapy increases stromal cell oxidative stress and DNA damage, which, in turn, promotes fibroblast activation and the secretion of senescence-associated secretory phenotypes (SASP). Several attempts at targeting CAF and stroma in PDAC have previously been unsuccessful. Targeting sonic hedgehog (SHH) or α-SMA has led to depletion of quiescent CAFs and promoted a more aggressive tumor biology [35–37]. Similarly, it has been shown in several murine cancer models, including PDAC, that targeting the dense desmoplasia relieves the suppressive effects on angiogenesis; therefore, facilitating the rebound growth of aberrant tumor vessels and promoting hypoxia, which contributed to the failure of this class of agents [38–41]. Our data demonstrates that ProAgio addresses these limitations. ProAgio plus chemotherapy treatment reprogrammed active CAFs to qCAFs rather than eliminating CAFs. This reprogramming leads to decreased secretion of metabolites, including glycine and cysteine, which support PDAC growth [42]. The decline of glycine and cysteine impairs the redox equilibrium and the biosynthetic ability of PDAC cells, contributing to the reduction of PDAC stemness and the expression of related genes [43, 44]. Furthermore, cysteine contributes to PDAC resistance to ferroptosis [45, 46].

Activated CAFs also contribute to PDAC progression, resistance, stemness, and immune evasion [47, 48]. ProAgio, in combination with chemotherapy, significantly modulated the immune profile in the orthotopic model and KPC-GEM model. scRNA-seq analysis showed an increase in CD8⁺ cytotoxic T cells in gemcitabine plus ProAgio-treated tumors. Flow cytometry data showed elevated levels of NK cells, CD8⁺, and CD4⁺ cells in PDAC-TME in combination therapy. Simultaneously, macrophages were polarized from an M2 to an M1 phenotype, indicating a pro-inflammatory response and a corresponding reduction in the tumor cell niche. ProAgio decreases CD4^+^ Treg and MDSCs, increases CD8^+^ cytotoxic T cells, and promotes M1 polarization. Upon activation, T-cells upregulate inhibitory checkpoint molecules such as PD-1 and CTLA-4. Higher expression of these molecules, in the presence of elevated IFN-γ, Granzyme-B, and perforin, indicates the activation phase. However, persistent TCR stimulation leads to upregulation of TIGIT, TIM-3, and Lag-3, which are regulatory molecules but may not downregulate IFN-γ, granzyme B, and perforin, which serve to prevent hyperactive immune responses. In our study, the presence of T cells with elevated expression of PD-1 and CTLA-4, with other immunoregulatory checkpoint molecules, suggests an activated phenotype as opposed to terminally exhausted T cells that have a similar expression pattern with loss of IFN- γ and other effector molecules. In sum, GPTx+ProAgio and FOI+ProAgio regimens both enhance sustained T cell activation and maintain expression of effector molecules that contribute to enhanced anti-tumor immunity.

### Limitations of the study

The experimental limitations of the in vivo setup, combined with the relatively small cohort size (*n* = 5 to 10 animals per group), significantly limit the statistical power. The consistency in the activity observed across multiple models does support the conclusion related to the enhanced activity of the combination treatment (Suppl. Table 4). Our group previously reported that when ProAgio binds to integrin β_3_, it induces apoptosis through activation of caspase-8 [11]. In our current study, we did observe a decrease in the integrin β_3_ expressing cells after treatment with ProAgio. A limitation of the current study is that we did not show that the decrease in integrin β_3_ expressing cells is directly linked to activation of caspase-8. Therefore, there is possibility that mechanisms other than activation of caspase-8 are linked to the observed decrease in integrin β_3_ expressing cells seen with ProAgio. The enhancement of the metastatic programs of KPC cells through culturing in conditioned medium was used to generate the mKPC-GB-Luc cell line used in this paper. Our data ensures no cross-species contamination occurred. The acquired metastatic phenoptype was maintained even after growth in the typical cell culture conditions (10% FBS in DMEM), implying that the phenotype exhibited by the cells is due to stable adaptation to metastasis and not transient signaling. This was further reinforced when our results were independently verified using the syngeneic metastatic mouse model KPC-ML1-Luc. Whether the mKPC-GB-Luc cell line is physiologically representative of spontaneous PDAC metastasis was not validated in this paper and is a limitation of this model. The demonstration of similar effects of ProAgio and chemotherapy in different mouse models in part addresses this limitation. Another limitation is related to differences in administration of cytotoxic chemotherapy in the mouse model compared to the clinical setting. In the case of FOLFIRINOX, 5-FU is administered as a continuous infusion clinically which cannot be replicated in a mouse model. Similarly, the regimen tested in our study did not include leucovorin. The data in this study clearly proves that ProAgio can improve the tumor inhibition of 5FU, Oxaliplatin and Irinotecan combination whether the same effects will be observed with FOLFIRINOX regimen will need to be eventually tested in future clinical trials. The brief length of the orthotopic model limits the development of tumor, driven stromal remodeling, and that the CAF states observed. To address this limitation, we also evaluated the combination in KPC GEM models which have a greater CAF diversification more comparable to human PDAC.

## Conclusion

Altogether, these results reveal the unique effects of ProAgio on the PDAC-TME by targeting both activated CAF and angiogenesis. ProAgio modulates hypoxia, metabolism, and immune activity in PDAC-TME. These findings support the continued clinical development of ProAgio and its evaluation with immune therapy in preclinical models.

## Supplementary Information

Supplementary Material 1: Fig. S1 mKPC-GB-Luc cells validation. A). Transcriptomic profile comparison between mKPC-GB-Luc cell lines and KPC-ML1-Luc cell line. B). Transcriptomic analysis revealed that most angiogenic markers were upregulated in mKPC-GB-Luc. C). Gene Ontology (GO) terms for biological processes and molecular processes (D).

Supplementary Material 2: Fig. S2 Chemotherapy plus ProAgio on PDAC tumor growth and CAFs reprogramming in the mKPC-GB-PDAC orthotopic mouse model. A). The body weight of each mouse in the study was measured every 5 days following orthotopic tumor implantation and continuing until study termination. B). Tumors were shown under various treatment conditions (sham, ProAgio, FOI, and FOI+ProAgio). C). H&E analysis reveals that FOI+ProAgio and other treatments, such as ProAgio and FOI, decrease the number of mitotic bodies in mKPC-GB-Luc tumors. D). ProAgio suppresses transforming growth factor beta (TGFβ) activation of pancreatic stellate cells (PSCs) E). Multiparameter flow cytometric analysis of CAFs showing subpopulations of CAFs in ex vivo tumor tissue from the mKPC-GB-Luc orthotopic murine model of PDAC. Bar 50 μm; One-way ANOVA, followed by Tukey’s multiple comparisons test, considered p values. ∗∗∗∗*p* <0.0001.

Supplementary Material 3: Fig. S3 Multiparameter FACS gating strategy for T, NK, and ICS panel (A). and myeloid panel (B).

Supplementary Material 4: Fig. S4 The combination of chemotherapy plus ProAgio on immune activation in the mKPC-GB-Luc orthotopic murine model of PDAC. A). Multiparameter flow cytometric analysis of CD45^+^ cells in ex vivo tumor tissue from the mKPC-GB-Luc orthotopic murine model of PDAC. B). Multiparameter flow cytometric analysis of macrophages showing a subpopulation of CD11b^+^ cells, F480^+^ macrophages, and M1/M2 ratio in ex vivo tumor tissue from the mKPC-GB-Luc orthotopic murine model. C-N). Multiparameter flow cytometric analysis of CD4^+^ Tregs, T cells, and intracellular cytokine staining showing the indicated T cell subpopulation and its functional status in ex vivo tumor tissue from the mKPC-GB-Luc orthotopic murine model. C). subpopulation of Tregs, D). subpopulation of CD4^+^ cells, E). subpopulation of CD8^+^ cells, F). subpopulation of CD4^+^ exhausted, effector, and central memory cells, G). subpopulation of CD8^+^ exhausted, effector, and central memory cells, H). subpopulation of TNK and NK cells I). subpopulation of γδ T delta cells, J). Intracellular cytokines CD4^+^ CD107α^+^, CD4^+^ TNFα^+^, and CD4^+^ INFγ^+^ cells in % of CD45 K). Intracellular cytokines CD8^+^ CD107α^+^, CD8^+^ TNFα^+^, CD8^+^ INFγ^+^ and CD8^+^ IL-17^+^ cells in % of CD45, L). Intracellular cytokines TNK^+^ CD107α^+^, TNK^+^ TNFα^+^, and TNK^+^ INFγ^+^ cells in % of CD45. Data is represented as mean ± SD. A one-way ANOVA test was used to analyze the data across treatment groups, where the data were normal. Nonparametric (Kruskal-Wallis). Tests equivalent to ANOVA were used when the parametric test assumptions were not met. Post-hoc tests comparing multiple groups were performed using Tukey’s test when using ANOVA and Dunn’s test when using Kruskal-Wallis. ns: non-significant, *p < 0.05, **p < 0.01, ***p < 0.001, and ****p < 0.0001.

Supplementary Material 5: Fig. S5 The combination of chemotherapy plus ProAgio on immune activation in the KPC-ML-1-Luc orthotopic murine model of PDAC. A). Multiparameter flow cytometric analysis of CD45^+^ cells in ex vivo tumor tissue from the KPC-ML1-Luc orthotopic murine model of PDAC. B). Multiparameter flow cytometric analysis of myeloid showing a subpopulation of CD11b^+^ cells, granulocytic MDSCs, monocytic MDSCss and M1/M2 ratio of macrophages. C). T and NK cell panels show a subpopulation of NK cells and γδ T cells D). CD4 compartment, including CD4^+^ T cells, Tregs, and exhausted CD4^+^ T cells. E). Subpopulation of CD4^+^ T cells F). CD8 compartment (CD8^+^ T cells, and exhausted CD8^+^ T cells) in the T and NK panel. G). Intracellular cytokines NK1.1^+^ CD107α^+^, INFγ^+^ , and granzyme B^+^ in % of CD45 H). Intracellular cytokines CD4^+^ CD107α^+^, TNFα^+^, INFγ^+^, and TNFα^+^, cells in % of CD45. I). Intracellular cytokines CD8^+^ CD107α^+^, TNFα^+^, INFγ^+^ and TNFα^+^ cells in % of CD45, J). Intracellular cytokines γδ^+^ T cells CD107α^+^, TNFα^+^, INFγ^+^ and TNFα^+^ cells in % of CD45. Data is represented as mean ± SD. A one-way ANOVA test was used to analyze the data across treatment groups, where the data were normal. Nonparametric (Kruskal-Wallis). Tests equivalent to ANOVA were used when the parametric test assumptions were not met. Post-hoc tests comparing multiple groups were performed using Tukey’s test when using ANOVA and Dunn’s test when using Kruskal-Wallis. ns: non-significant, *p < 0.05, **p < 0.01, ***p < 0.001, and ****p < 0.0001.

Supplementary Material 6: Fig. S6 Terminal tumor weight and liver metastasis spots. scRNA-Seq analysis shows a marked decrease in GEM-PDAC intratumoral cell populations in mice treated with Gem + ProAgio. A). Terminal tumor weight B). Liver metastatic spots C). Cell count and percentage of GEM-PDAC intratumoral cell populations in sham and Gem or ProAgio or Gem+ProAgio treatments. D). A dot plot displays migration & adhesion signaling E). autophagy signaling F). ER-stress signaling and G). Wnt signaling pathway gene markers.

Supplementary Material 7: Fig. S7 The combination of ProAgio with Gem on integrins, endoplasmic reticulum stress, autophagy, survival, migration, adhesion, angiogenesis, invasion, epithelial-mesenchymal, insulin, cytokine, growth factor, and stemness pathways in ADM clusters of the GEM-PDAC model. A-L). Dot plot analysis displays a reduction in combination treatment compared to other individual drug-treated mice, except for (S. Fig. 7H)—epithelial markers. Epithelial gene markers are upregulated in combination therapy.

Supplementary Material 8: Fig. S8 The combination of ProAgio with Gem on integrins, endoplasmic reticulum stress, autophagy, survival, angiogenesis, cytokine, and growth factor pathways in endothelial clusters of the GEM-PDAC model. A-G). Dot plot analysis displays a reduction in combination treatment compared to other individual drug-treated mice.

Supplementary Material 9: Fig. S9 Gemcitabine (G) with ProAgio modulates cytokines and growth factors in serum and tumor samples of GEM-PDAC models. A-C). VEGF, IGF1, and CXCL2 are significantly decreased in serum and tumor samples from Gem+ProAgio-treated GEM-PDAC models. D). No significant difference in IL-10 levels was observed between treatments in either serum or tumor samples. E). IL6 significantly increases in the serum of ProAgio or Gem+ProAgio-treated mice compared to G-treated animals. IL6 levels are higher in tumors from ProAgio- or Gem+ProAgio-treated than in sham-treated mice. F-I). Granulocyte macrophage colony-stimulating factor (GM-CSF), Granulocyte colony-stimulating factor (GCSF), Transforming growth factor beta 1 (TGFβ1), and Epidermal growth factor (EGF) are significantly decreased in serum and tumor samples from Gem+ProAgio-treated GEM-PDAC models compared to sham. Data is represented as mean ± SD. A one-way ANOVA test was used to analyze the data across treatment groups, where the data were normal. Nonparametric (Kruskal-Wallis). Tests equivalent to ANOVA are used when the assumptions of a parametric test are not met. Post-hoc tests comparing multiple groups were performed using Tukey’s test when using ANOVA and Dunn’s test when using Kruskal-Wallis. ns: non-significant, *p < 0.05, **p < 0.01, ***p < 0.001, and ****p < 0.0001.

Supplementary Material 10.

Supplementary Material 11.

Supplementary Material 12.

## Data Availability

The sequencing data generated in this study (bulk RNA-seq and scRNA) were deposited in the GEO repository under project accession numbers GSE312137 (GSM9338303, GSM9338304, GSM9338305, GSM9338306, GSM9338307, and GSM9338308) and GSE312138 (GSM9338309). The remaining data are available within the article and its Supplementary Information or from the corresponding author on request. Source data are provided with this paper.

## Code availability

No new algorithms were developed for this study. All code generated for analysis is publicly available from GitHub https://github.com/Pganji7353/Gem_ProAgio_All_Codes.

## References

[1] Siegel RL, Kratzer TB, Giaquinto AN, Sung H, Jemal A. Cancer statistics, 2025. CA Cancer J Clin. 2025;75:10. 10.3322/caac.21871 PMC11745215 39817679

[2] Maddipati R. Metastatic heterogeneity in pancreatic cancer: mechanisms and opportunities for targeted intervention. J Clin Investig. 2025;135:e191943. 10.1172/JCI191943 PMC12259258 40662361

[3] Zhou Y, Ma Y, Sheng J, Ma Y, Ding J, Zhou W. Breaking down barriers in drug delivery by stromal remodeling approaches in pancreatic cancer. Mol Pharm. 2024;21:3764–76. 10.1021/acs.molpharmaceut.4c00329 39049481

[4] Wu C, Von Schalscha T, Sansanwal D, Qian C, Jiang Q, Shepard RM, Wettersten HI, McCormack SJ, Weis SM, Cheresh DA. Targeting pancreatic cancer cell stemness by blocking fibronectin-binding integrins on cancer-associated fibroblasts. Cancer Res Commun. 2025;5:195–208. 10.1158/2767-9764.CRC-24-0491 PMC11783622 39785683

[5] Pednekar K, Heinrich MA, Prakash J. Role of integrins in the tumor stroma. The Tumor Stroma. Jenny Stanford Publishing. 2022;233–86. 10.1201/9781003224921.

[6] Nakamoto R, Ferri V, Duan H, Hatami N, Goel M, Rosenberg J, Kimura R, Wardak M, Haywood T, Kellow R. Pilot-phase PET/CT study targeting integrin αvβ6 in pancreatic cancer patients using the cystine-knot peptide–based 18F-FP-R01-MG-F2. Eur J Nucl Med Mol Imaging. 2022;50:184–93. 10.1007/s00259-021-05595-7 34729628

[7] Li S, Sampson C, Liu C, Piao H-l, Liu H-X. Integrin signaling in cancer: bidirectional mechanisms and therapeutic opportunities. Cell Communication Signal. 2023;21:266. 10.1186/s12964-023-01264-4 PMC10537162 37770930

[8] Nan P, Dong X, Bai X, Lu H, Liu F, Sun Y, Zhao X. Tumor-stroma TGF-β1-THBS2 feedback circuit drives pancreatic ductal adenocarcinoma progression via integrin αvβ3/CD36-mediated activation of the MAPK pathway. Cancer Lett. 2022;528:59–75. 10.1016/j.canlet.2021.12.025 34958892

[9] Guo D-y, Chen Z-h, Fu Y-f, Li Y-y, Chen M-n, Wu J-j, Yuan Z-d, Ye J-X, Li X. Yuan F-l: Cilengitide inhibits osteoclast adhesion through blocking the αvβ3-mediated FAK/Src signaling pathway. Heliyon. 2023;9:e17841. 10.1016/j.heliyon.2023.e17841 PMC10395300 37539209

[10] Campos AD, Shepard RM, Ortega Z, Heumann I, Wilke AE, Nam A, Cable C, Kouhmareh K, Klemke R, Mattson NM. A STAT3/integrin axis accelerates pancreatic cancer initiation and progression. Cell Rep. 2025;44:116010. 10.1016/j.celrep.2025.116010 PMC12445075 40701148

[11] Turaga RC, Sharma M, Mishra F, Krasinskas A, Yuan Y, Yang JJ, Wang S, Liu C, Li S, Liu Z-R. Modulation of cancer-associated fibrotic stroma by an integrin αvβ3 targeting protein for pancreatic cancer treatment. Cell Mol Gastroenterol Hepatol. 2021;11:161–79. 10.1016/j.jcmgh.2020.08.004 PMC7674520 32810598

[12] Turaga RC, Yin L, Yang JJ, Lee H, Ivanov I, Yan C, Yang H, Grossniklaus HE, Wang S, Ma C. Rational design of a protein that binds integrin αvβ3 outside the ligand binding site. Nat Commun. 2016;7:11675. 10.1038/ncomms11675 PMC4895024 27241473

[13] Turaga RC, Satyanarayana G, Sharma M, Yang JJ, Wang S, Liu C, Li S, Yang H, Grossniklaus H, Farris AB. Targeting integrin αvβ3 by a rationally designed protein for chronic liver disease treatment. Commun Biology. 2021;4:1087. 10.1038/s42003-021-02611-2 PMC8445973 34531529

[14] Sharma M, Turaga RC, Yuan Y, Satyanarayana G, Mishra F, Bian Z, Liu W, Sun L, Yang J, Liu Z-R. Simultaneously targeting cancer-associated fibroblasts and angiogenic vessel as a treatment for TNBC. J Exp Med. 2021;218:e20200712. 10.1084/jem.20200712 PMC7876552 33561195

[15] Horvat NK, Karpovsky I, Phillips M, Wyatt MM, Hall MA, Herting CJ, Hammons J, Mahdi Z, Moffitt RA, Paulos CM. Clinically relevant orthotopic pancreatic cancer models for adoptive T cell transfer therapy. J Immunother Cancer. 2024;12:e008086. 10.1136/jitc-2023-008086 PMC10806555 38191243

[16] Khaliq AM, Rajamohan M, Saeed O, Mansouri K, Adil A, Zhang C, Turk A, Carstens JL, House M, Hayat S. Spatial transcriptomic analysis of primary and metastatic pancreatic cancers highlights tumor microenvironmental heterogeneity. Nat Genet. 2024;56:2455–65. 10.1038/s41588-024-01914-4 39294496

[17] Dong D, Zhou Z, Zhu M, Hou Z, Chen M, Gong J, Zhao X, Yan A, Liang H, Yin Y. STN1 facilitates metastasis by promoting transcription of EMT-activator ZEB1 in pancreatic cancer. Nat Commun. 2025;16:7815. 10.1038/s41467-025-63083-0 PMC12370990 40841373

[18] Pérez-Gutiérrez L, Ferrara N. Biology and therapeutic targeting of vascular endothelial growth factor A. Nat Rev Mol Cell Biol. 2023;24:816–34. 10.1038/s41580-023-00631-w 37491579

[19] Öhlund D, Handly-Santana A, Biffi G, Elyada E, Almeida AS, Ponz-Sarvise M, Corbo V, Oni TE, Hearn SA, Lee EJ. Distinct populations of inflammatory fibroblasts and myofibroblasts in pancreatic cancer. J Exp Med. 2017;214:579–96. 10.1084/jem.20162024 PMC5339682 28232471

[20] Elyada E, Bolisetty M, Laise P, Flynn WF, Courtois ET, Burkhart RA, Teinor JA, Belleau P, Biffi G, Lucito MS. Cross-species single-cell analysis of pancreatic ductal adenocarcinoma reveals antigen-presenting cancer-associated fibroblasts. Cancer Discov. 2019;9:1102–23. 10.1158/2159-8290.CD-19-0094 PMC6727976 31197017

[21] Nagaraju GP, Saddala MS, Foote JB, Khaliq AM, Masood A, Golivi Y, Bandi DSR, Sarvesh S, Reddy SP, Switchenko J. Mechanism of enhancing chemotherapy efficacy in pancreatic ductal adenocarcinoma with paricalcitol and hydroxychloroquine. Cell Rep Med. 2025;6:101881. 10.1016/j.xcrm.2024.101881 PMC11866435 39730001

[22] Schwörer S, Cimino FV, Ros M, Tsanov KM, Ng C, Lowe SW, Carmona-Fontaine C, Thompson CB. Hypoxia potentiates the inflammatory fibroblast phenotype promoted by pancreatic cancer cell–derived cytokines. Cancer Res. 2023;83:1596–610. 10.1158/0008-5472.CAN-22-2316 PMC10658995 36912618

[23] Mello AM, Ngodup T, Lee Y, Donahue KL, Li J, Rao A, Carpenter ES, Crawford HC, di Pasca M, Lee KE. Hypoxia promotes an inflammatory phenotype of fibroblasts in pancreatic cancer. Oncogenesis. 2022;11:56. 10.1038/s41389-022-00434-2 PMC9478137 36109493

[24] Sazeides C, Le A. Metabolic relationship between cancer-associated fibroblasts and cancer cells. The heterogeneity of cancer metabolism. Springer International Publishing Cham, 2021;1311:189–204. 10.1007/978-3-030-65768-0_14 PMC9703248 34014544

[25] Encarnacion-Rosado J, Kimmelman AC. Harnessing metabolic dependencies in pancreatic cancers. Nat Reviews Gastroenterol Hepatol. 2021;18:482–92. 10.1038/s41575-021-00431-7 PMC8249349 33742165

[26] Chen Z, Han F, Du Y, Shi H, Zhou W. Hypoxic microenvironment in cancer: molecular mechanisms and therapeutic interventions. Signal Transduct Target therapy. 2023;8:70. 10.1038/s41392-023-01332-8 PMC9935926 36797231

[27] Yang J, Liu Y, Liu S. The role of epithelial-mesenchymal transition and autophagy in pancreatic ductal adenocarcinoma invasion. Cell Death Dis. 2023;14:506. 10.1038/s41419-023-06032-3 PMC10406904 37550301

[28] Giannoni E, Parri M, Chiarugi P. EMT and oxidative stress: a bidirectional interplay affecting tumor malignancy. Antioxid Redox Signal. 2012;16:1248–63. 10.1089/ars.2011.4280 21929373

[29] Sun JD, Liu Q, Ahluwalia D, Li W, Meng F, Wang Y, Bhupathi D, Ruprell AS, Hart CP. Efficacy and safety of the hypoxia-activated prodrug TH-302 in combination with gemcitabine and nab-paclitaxel in human tumor xenograft models of pancreatic cancer. Cancer Biol Ther. 2015;16:438–49. 10.1080/15384047.2014.1003005 PMC4623012 25679067

[30] Li Q, Yang C, Li J, Wang R, Min J, Song Y, Su H. The type I collagen paradox in PDAC progression: microenvironmental protector turned tumor accomplice. J Translational Med. 2025;23:744. 10.1186/s12967-025-06778-8 PMC12228414 40616159

[31] Arora S, Bhardwaj A, Singh S, Srivastava SK, McClellan S, Nirodi CS, Piazza GA, Grizzle WE, Owen LB, Singh AP. An undesired effect of chemotherapy: gemcitabine promotes pancreatic cancer cell invasiveness through reactive oxygen species-dependent, nuclear factor κB-and hypoxia-inducible factor 1α-mediated up-regulation of CXCR4. J Biol Chem. 2013;288:21197–207. 10.1074/jbc.M113.484576 PMC3774385 23740244

[32] Abdalla MY, Ahmad IM, Rachagani S, Banerjee K, Thompson CM, Maurer HC, Olive KP, Bailey KL, Britigan BE, Kumar S. Enhancing responsiveness of pancreatic cancer cells to gemcitabine treatment under hypoxia by heme oxygenase-1 inhibition. Translational Res. 2019;207:56–69. 10.1016/j.trsl.2018.12.008 30653942

[33] Rebelo R, Xavier CP, Giovannetti E, Vasconcelos MH. Fibroblasts in pancreatic cancer: molecular and clinical perspectives. Trends Mol Med. 2023;29:439–53. 10.1016/j.molmed.2023.03.002 37100646

[34] Melchionna R, Trono P, Di Carlo A, Di Modugno F, Nisticò P. Transcription factors in fibroblast plasticity and CAF heterogeneity. J Experimental Clin Cancer Res. 2023;42:347. 10.1186/s13046-023-02934-4 PMC10731891 38124183

[35] Özdemir BC, Pentcheva-Hoang T, Carstens JL, Zheng X, Wu C-C, Simpson TR, Laklai H, Sugimoto H, Kahlert C, Novitskiy SV. Depletion of carcinoma-associated fibroblasts and fibrosis induces immunosuppression and accelerates pancreas cancer with reduced survival. Cancer Cell. 2014;25:719–34. 10.1016/j.ccr.2014.04.005 PMC4180632 24856586

[36] Rhim AD, Oberstein PE, Thomas DH, Mirek ET, Palermo CF, Sastra SA, Dekleva EN, Saunders T, Becerra CP, Tattersall IW. Stromal elements act to restrain, rather than support, pancreatic ductal adenocarcinoma. Cancer Cell. 2014;25:735–47. 10.1016/j.ccr.2014.04.021 PMC4096698 24856585

[37] Leake I. Surprising role for fibrosis. Nat Reviews Gastroenterol Hepatol. 2014;11:396–396. 10.1038/nrgastro.2014.97 24912392

[38] Longo V, Brunetti O, Gnoni A, Cascinu S, Gasparini G, Lorusso V, Ribatti D, Silvestris N. Angiogenesis in pancreatic ductal adenocarcinoma: A controversial issue. Oncotarget. 2016;7:58649. 10.18632/oncotarget.10765 PMC5295459 27462915

[39] Cannon A, Thompson C, Hall BR, Jain M, Kumar S, Batra SK. Desmoplasia in pancreatic ductal adenocarcinoma: insight into pathological function and therapeutic potential. Genes cancer. 2018;9:78. 10.18632/genesandcancer.171 PMC6086006 30108679

[40] Jacobetz MA, Chan DS, Neesse A, Bapiro TE, Cook N, Frese KK, Feig C, Nakagawa T, Caldwell ME, Zecchini HI. Hyaluronan impairs vascular function and drug delivery in a mouse model of pancreatic cancer. Gut. 2013;62:112–20. 10.1136/gutjnl-2012-302529 PMC3551211 22466618

[41] Tamburrino A, Piro G, Carbone C, Tortora G, Melisi D. Mechanisms of resistance to chemotherapeutic and anti-angiogenic drugs as novel targets for pancreatic cancer therapy. Front Pharmacol. 2013;4:56. 10.3389/fphar.2013.00056 PMC3639407 23641216

[42] Sazeides C, Le A. Metabolic relationship between cancer-associated fibroblasts and cancer cells. Heterogeneity Cancer Metabolism. 2018;1063:149–65. 10.1007/978-3-319-77736-8_11 29946782

[43] Ortiz AG. Assessing the phenotype of pancreatic cancer stem cells under adaptation to serine and glycine deficiency. Effects on metabolic plasticity and immune evasive properties. 2024;1–26. Undergraduate thesis.

[44] Ogunleye AO, Nimmakayala RK, Batra SK, Ponnusamy MP. Metabolic rewiring and stemness: a critical attribute of pancreatic cancer progression. Stem Cells. 2023;41:417–30. 10.1093/stmcls/sxad017 PMC10183971 36869789

[45] Badgley MA, Kremer DM, Maurer HC, DelGiorno KE, Lee H-J, Purohit V, Sagalovskiy IR, Ma A, Kapilian J, Firl CE. Cysteine depletion induces pancreatic tumor ferroptosis in mice. Science. 2020;368:85–9. 10.1126/science.aaw9872 PMC7681911 32241947

[46] Zhu Y, Fang S, Fan B, Xu K, Xu L, Wang L, Zhu L, Chen C, Wu R, Ni J. Cancer-associated fibroblasts reprogram cysteine metabolism to increase tumor resistance to ferroptosis in pancreatic cancer. Theranostics. 2024;14:1683. 10.7150/thno.89805 PMC10879865 38389839

[47] Yamamoto Y, Kasashima H, Fukui Y, Tsujio G, Yashiro M, Maeda K. The heterogeneity of cancer-associated fibroblast subpopulations: Their origins, biomarkers, and roles in the tumor microenvironment. Cancer Sci. 2023;114:16–24. 10.1111/cas.15609 PMC9807521 36197901

[48] Mizutani Y, Kobayashi H, Iida T, Asai N, Masamune A, Hara A, Esaki N, Ushida K, Mii S, Shiraki Y. Meflin-positive cancer-associated fibroblasts inhibit pancreatic carcinogenesis. Cancer Res. 2019;79:5367–81. 10.1158/0008-5472.CAN-19-0454 31439548

