# Supplementary material for: Reprogramming the pancreatic ductal adenocarcinoma microenvironment: a novel integrin-targeted cytotoxin, ProAgio, potentiates chemotherapy
Source: Mol Cancer. 2026 Jul 3;25:233. doi: 10.1186/s12943-026-02725-3 (PMC13643974; doi:10.1186/s12943-026-02725-3)
Supplement: Supplementary file 1 [file 12943_2026_2725_MOESM12_ESM.docx]

**Suppl. Table 4.** Tumor weights and percent change.

| **Model** | **Experiment** | **Sham** | **Chemotherapy** | **ProAgio** | **Chemotherapy + ProAgio** |
| --- | --- | --- | --- | --- | --- |
| mKPC-GB-Luc  (n=5; IP) | FOI+ProAgio | 3.1 ± 0.46 | 1.3 ± 0.11  (-58.1%) | 1.4 ± 0.37  (-54.8%) | 0.5 ± 0.16  (-83.9%) |
| KPC-ML1-Luc  (n=10; IV) | GPTx+ProAgio | 3.8 ± 0.36 | 2.99 ± 0.52  (-21.3%) | 2.84 ± 0.56  (-25.3%) | 1.0 ± 0.30  (-67.7%) |
| KPC-ML1-Luc  in Rag KO  (n=9; IV) | GPTx+ProAgio | 3.56 ± 0.28 |  |  | 3.13 ± 0.58  (-12.1%) |
| GEM model  (n=5; IP) | Gem+ProAgio | 3.15 ± 0.87 | 2.37 ± 0.96  (-24.8%) | 1.91 ± 0.59  (-39.4%) | 0.58 ± 0.22  (-81.6%) |

Summary of the experiments presented in the paper. The data represents the mean tumor weights (g) at the end of the experiment with standard deviation. In addition, the percent change from sham is shown for chemotherapy, ProAgio and combination.

Values are mean ± S.D.

Values in parentheses are percentage change from the sham group.
