# Supplementary material for: Reprogramming the pancreatic ductal adenocarcinoma microenvironment: a novel integrin-targeted cytotoxin, ProAgio, potentiates chemotherapy
Source: Mol Cancer. 2026 Jul 3;25:233. doi: 10.1186/s12943-026-02725-3 (PMC13643974; doi:10.1186/s12943-026-02725-3)
Supplement: Supplementary file 2 [file 12943_2026_2725_MOESM10_ESM.doc]

**Materials and Methods**

**Cell lines**

The murine PDAC cell lines (RRID:CVCL_0480), mKPC-GB-Luc (RRID:CVCL_UY60), and KPC-Luc (RRID:CVCL_UY60), pancreatic stellate cells (PSCs; RRID:CVCL_A5TH), and the human PDAC cell line PANC-1 were maintained as explained in our previous publications [1] and ATCC instructions (American Type Culture Collection, VA, USA). Prof. Gregory B. Lesinski, Emory University, USA, gifted the KPC-Luc cell line. Dr. Denis C Guttridge, Medical University of South Carolina, USA, gifted the KPC-ML1-Luc cell line. KPC cell lines were assessed for a comprehensive panel of pathogens, with mycoplasma, prior to animal studies, in accordance with the University of Alabama IACUC and ARP procedures. All materials, antibodies, and reagents details were given in Supplementary Table 1.

Supplementary Table 1 Details about reagents, resources, and identifiers.

| **REAGENT or RESOURCE** | **SOURCE** | **IDENTIFIER** |
| --- | --- | --- |
| **Antibodies & Clones** |  |  |
| PD-1 FITC & 29F.1A12 | BioLegend | Cat#135214; RRID:AB_10680238 |
| CD206 FITC & C068C2 | BioLegend | Cat#141703; RRID:AB_10900988 |
| CD107α & D4B | BioLegend | Cat#121607; RRID:AB_571982 |
| TIM-3 PE & B8.2C12 | BioLegend | Cat#134003; RRID:AB_1626181 |
| F4/80 PE & BM8 | BioLegend | Cat#123110; RRID:AB_893486 |
| IL-17F PE & 9D3.1C8 | BioLegend | Cat#517008; RRID:AB_10690818 |
| CD19 PE Cy7 & 1D3/CD19 | BioLegend | Cat#152417; RRID:AB_2927870 |
| Granzyme B PE Cy7 & QA16A02 | BioLegend | Cat#372214; RRID:AB_2728381 |
| Ly6C PE Cy7 & HK1.4 | BioLegend | Cat#128017; RRID:AB_1732093 |
| CD11 b PE Dazzle & M1/70 | BioLegend | Cat#101255; RRID:AB_2563647 |
| IFNγ PE Dazzle & XMG1.2 | BioLegend | Cat#505846; RRID:AB_2563980 |
| CD45 PerCP & 30-F11 | BioLegend | Cat#103130; RRID:AB_893339 |
| CD11c APC & N418 | BioLegend | Cat#117309; RRID:AB_313778 |
| TNFα APC & MP6-XT22 | BioLegend | Cat#506308; RRID:AB_315429 |
| CD172α Alexa Flour 700 & P84 | BioLegend | Cat#144022; RRID:AB_2650813 |
| CD62L APC Cy7 & MEL-14 | BioLegend | Cat#104428; RRID:AB_830799 |
| CD86 APC Cy7 & GL-1 | BioLegend | Cat#105045; RRID:AB_2629769 |
| IL-2 APC 750 & JES6-5H4 | BioLegend | Cat#503832; RRID:AB_2561750 |
| CD3ε BV421&145-2C11 | BioLegend | Cat#100341; RRID:AB_2562556 |
| XCR1 BV421& ZET | BioLegend | Cat#148216; RRID:AB_2565230 |
| Ly6G BV570 &1A8 | BioLegend | Cat#127629; RRID:AB_10899738 |
| TCR γ/δ BV 605 & GL3 | BioLegend | Cat#118129; RRID:AB_2563356 |
| PD-L1 BV 605 & MIH6 | BioLegend | Cat#153606; RRID:AB_2814056 |
| CD4 BV 650 & GK1.5 | BioLegend | Cat#100469; RRID:AB_2783035 |
| MHCII BV 650 & M5/114.15.2 | BioLegend | Cat#107641;RRID:AB_2565975 |
| NK1.1 BV 711& S17016D | BioLegend | Cat#156541; RRID:AB_3097465 |
| CD103 BV 711& 2E7 | BioLegend | Cat#121435; RRID:AB_2686970 |
| CD8a BV 785 & 53-6.7 | BioLegend | Cat#100750; RRID:AB_2562610 |
| CCR7 BV785 & G043H7 | BioLegend | Cat# 353230; RRID:AB_2563630 |
| Fc Block (CD16/CD32) & 93 | BioLegend | Cat#101320; RRID:AB_1574975 |
| Thy1.1 PE & OX-7 | BD Biosciences | Cat#551401;RRID:AB_394185 |
| CTLA-4 APCR700 & UC10-4F10-11 | BD Biosciences | Cat#565778; RRID:AB_2739350 |
| CD44 BUV737 & IM7 | BD Biosciences | Cat#612799; RRID:AB_2870126 |
| CD69 BUV395 & H1.2F3 | BD Biosciences | Cat#740220; RRID:AB_2739968; |
| CD45R/B220 BUV395 & Q38-480 | BD Biosciences | Cat#563793;RRID:AB_2738427 |
| Foxp3 APC & FJK-16s | Thermo Fisher Scientific | Cat#17-5773-82; RRID:AB_469457 |
| Ki67 & D3B5 | Cell signaling technology | Cat#12202; RRID:AB_2620142 |
| HIF-1α & Polyclonal | R and D Systems | Cat#A1935; RRID:AB_355064 |
| Integrin beta 3 & M581 | Thermo Fisher Scientific | Cat#MA5-47721; RRID:AB_2942702 |
| E-Cadherin & Polyclonal | Proteintech | Cat#20874-1-AP; RRID:AB_10697811 |
| CD8a & 4SM16 | Thermo Fisher Scientific | Cat#14-0195-80; RRID:AB_2637158 |
| CD31 & D8V9E | Cell signaling technology | Cat#77699S;RRID:AB_2722705 |
| Col1A1& Polyclonal | LSBio | Cat#LS-C783682 |
| Podoplanin & eBio8.1.1 (8.1.1) | Thermo Fisher Scientific | Cat#14-5381-82; RRID:AB_1210505 |
| VEGF-A & EP1176Y | Abcam | Cat#AB52917; RRID:AB_883427 |
| pEGFR & EGFRY1068-E5 | Thermo Fisher Scientific | Cat#MA5-36966;RRID:AB_2896901 |
| CD24-APC & 30-F1 | BioLegend | Cat#138506; RRID:AB_2565651 |
| CD133-BV421 & 315-2C11 | BioLegend | Cat#141213; RRID:AB_2566011 |
| PAB2627 (Hypoxprobe) | Chromaprobe | Cat#R2710-A |
| Live/Dead Aqua | Thermo Fisher Scientific | Cat#L34966 |
|  |  |  |
| AlexaFluor568 anti-Rabbit | Thermo Fisher Scientific | Cat# A-11011;RRID:AB_143157 |
| Anti-rabbit IgG, HRP-linked Antibody | Cell signaling technology | Cat#7074;RRID:AB_2099233 |
| Anti-mouse IgG, HRP-linked Antibody | Cell signaling technology | Cat#7076; RRID:AB_330924 |
| AlexaFluor488 anti-Goat | Thermo Fisher Scientific | Cat#A11055;RRID:AB_2534102 |
| AlexaFluor488 anti-Rabbit | Thermo Fisher Scientific | Cat#A11008;RRID:AB_143165 |
| AlexaFluor568 anti-Mouse | Thermo Fisher Scientific | Cat#A11004;RRID:AB_2534072 |
| AlexaFluor647 anti-Hamster | Thermo Fisher Scientific | Cat#A21451;AB_2535868 |
| AlexaFluor568 anti-Rat | Thermo Fisher Scientific | Cat#A11077;RRID:AB_2534121 |
| AlexaFluor647 anti-Goat | Thermo Fisher Scientific | Cat#A21447;RRID:AB_2535864 |
| AlexaFluor568 anti-Goat | Thermo Fisher Scientific | A11057; RRID:AB_2534104 |
| AlexaFluor488 anti-Mouse | Thermo Fisher Scientific | A11001;RRID:AB_2534069 |
| AlexaFluor647 anti-rat | Thermo Fisher Scientific | A21247;RRID:AB_141778 |
| AlexaFluor647 anti-Mouse | Thermo Fisher Scientific | A21236; RRID:AB_2535805 |
| AlexaFluor647 anti-Rabbit | Thermo Fisher Scientific | A21244; RRID:AB_2535812 |
| AlexaFluor488 anti-Rat | Thermo Fisher Scientific | A21208; RRID:AB_2535794 |
|  |  |  |
|  |  |  |
| **Chemicals, peptides, and recombinant proteins** | |  |
| Matrigel Matrix | Corning | Cat#354234 |
| RBC lysis buffer | Thermo Scientific | Cat#J62150.AK |
| BSA | Fisher Scientific | Cat#BP1600 |
| Liberase | Roche | Cat#05401127001 |
| Hematoxylin solution | Sigma-Aldrich | Cat#51275 |
| Eosin | Millipore Sigma | Cat#1737287-1 |
| collagenase D | Millipore Sigma | Cat#11088858001 |
| DNase I | Millipore Sigma | Cat#10104159001 |
| DMEM | ATCC | Cat#30-2002 |
| Penicillin-Streptomycin | ATCC | Cat#30-2300 |
| PBS | ATCC | Cat#30-2200 |
| Trypsin-EDTA | ATCC | Cat#30-2101 |
| FBS | Gibco | Cat#16170-078 |
|  |  |  |
| **Critical commercial assays** |  |  |
| Vectastain Elite ABC Universal PLUS Kit | Vector | Cat#PK-8200 |
| NextGem 3′V3.1 kit | 10X Genomics | Cat#CG000204 |
| Mouse VEGF Quantikine ELISA Kit | R&D Systems | Cat#MMV00-1 |
| MouseIGF-I/IGF-1 Quantikine ELISA Kit | R&D Systems | Cat#SMG100 |
| Mouse CXCL2/MIP-2 Quantikine ELISA Kit | R&D Systems | Cat#SMM200 |
| Mouse IL-10 Quantikine ELISA Kit | R&D Systems | Cat#SM1000B |
| Mouse IL-6 Quantikine ELISA Kit | R&D Systems | Cat#SM6000B |
| Mouse GM-CSF Quantikine ELISA Kit | R&D Systems | Cat#SMGM00 |
| Mouse G-CSF Quantikine ELISA Kit | R&D Systems | Cat#MCS00 |
| Human/Mouse/Rat/Porcine/Canine TGF-beta 1 Quantikine ELISA | R&D Systems | Cat#SB100C |
| Mouse EGF Quantikine ELISA Kit | R&D Systems | Cat#MEG00 |
| **Deposited data** |  |  |
| Bulk RNA-seq | This paper | Acc#GSE312137 |
| scRNA-seq raw data deposited in GEO | This paper | Acc#GSE312138 |
| Code pipeline deposited in Github | This paper | [https://github.com/Pganji7353/Gem_ProAgio_All_Codes](https://urldefense.com/v3/__https:/github.com/Pganji7353/Gem_ProAgio_All_Codes__;!!NoSwA-eRAg!BBhu5e69W1GZwzfgZ1CWlWMWynteYg67xrUznAlGR5CNQdEFfkqid3iaD7YPCaeIwmTlXh5ZeJ1AyEYnUXQsGYWt$) |
|  |  |  |
| **Experimental models: Cell lines** |  |  |
| PANC-1 | ATCC | Cat#CRL-1469; RRID:CVCL_0480 |
| KPC_Luc cells | This paper | RRID:CVCL_UY60 |
| mKPC-GB-Luc cells | This paper |  |
| Pancreatic stellate cells | This paper |  |
| KPC-ML1-Luc cells | This paper |  |
| **Experimental models: Organisms/strains** | |  |
| C57BL/6J male mouse | Jackson Labs | Strain #:000664; RRID:IMSR_JAX:000665 |
| Kras^LSL-G12D^;p53^LoxP^;Pdx1-CreER | Jackson Labs | Strain #:032429; RRID:IMSR_JAX:032429 |
| LSL-K-ras G12D (B6N) | Jackson Labs | Strain #:019104; RRID:IMSR_JAX:019104 |
| p53LSL.R172H 129svj | Jackson Labs | Strain #:008652; RRID:IMSR_JAX:008652 |
| Pdx-Cre | Jackson Labs | Strain #:014647; RRID:IMSR_JAX:014647 |
| RagKO (B6.129S7-*Rag1^tm1Mom^*/J) | Jackson Labs | Strain #:002216; RRID:IMSR_JAX:002216 |
| **Recombinant DNA** |  |  |
| N/A |  |  |
|  |  |  |
| **Software and algorithms** |  |  |
| FlowJo version 10.7.2 | FlowJo, LLC | RRID:SCR_008520 |
| Graphpad Prism | GraphPad software | RRID:SCR_002798 |
| ImageJ | This paper | RRID:SCR_003070 |
| BioRender | HomeStars | RRID:SCR_018361 |
| Cell ranger 9.0.1 | 10X Genomics | RRID:SCR_017344 |
| Seurat (v.5.3.0) R package v.4.5.1. | Paul Hoffman, Satija | RRID#SCR_016341 |
|  | Lab and Collaborators |  |
| Python | Python Programming Language | RRID:SCR_008394 |
| Cellpose 3.0 | Cellpose | RRID:SCR_021716 |
| **Other** |  |  |
| Pimonidazole (hydrochloride) | MedChemExpress | Cat#HY-105129 |
| ProAgio (ACT50) | Xiamen Amoytop Biotech Co | NA |
| Gemcitabine | AM Beed | Cat#A433642 |
| 5-fluorouracil (5-FU) | Selleckchem | Cat#S1209 |
| Oxaliplatin | Selleckchem | Cat#S1224 |
| Irinotecan | Selleckchem | Cat#S1198 |
| Nab-Paclitaxel | Selleckchem | Cat#E1068 |

**Pancreatic stellate cell activation and sample preparation for HPLC**

A 6-well plate's 1 mL of conditioned medium from pancreatic stellate cells cultured in TGFβ (Cat # 7666-MB-005/CF), TGFβ+ProAgio, or no TGFβ was transferred to a tube and speed-vac at 37 °C until the volume was reduced by half. Trichloroacetic acid was used to deproteinize the samples, and 1M NaOH was used to neutralize them. After centrifugation, the samples were kept on ice for 20 minutes, and the supernatants were transferred to a 3000-MWCO filter and centrifuged at 10,000 × g for 30 minutes.

**Amino acid derivatization**

A series of amino acid standards was dried under a rotavap at 60 °C for 4 hours. The dried standards were then dissolved in the coupling solution (acetonitrile: pyridine: triethylamine: H_2_O (10:5:2:3)). 5 μL of phenylisothiocyanate (PITC) was added to the standards, and the mixture was incubated at RT for 5 minutes. They were then dried again under a rotavap at 60 °C for 4 hours. The dried pellets were reconstituted in 200 μL of 0.05M ammonium acetate. Serine and glycine stocks: HPLC-grade ultrapure water was used to create stock solutions of L-serine and L-glycine at concentrations of 0, 25, 50, 100, 200, and 500 pmol/μL.

**Chromatographic conditions**

The HPLC run conditions were as follows: solution A: 20 mM sodium acetate + 2 mg EDTA + 0.018% triethylamine (v/v), pH adjusted to 7.2. Solution B: methanol: acetonitrile 1:1 + 20 mM sodium acetate and pH adjusted to 7.2. The solutions were run with the following gradient:

| **Time (min)** | **%A** | **%B** |
| --- | --- | --- |
| 0 | 100 | 0 |
| 17 | 40 | 60 |
| 22 | 0 | 100 |
| 24 | 0 | 100 |
| 25 | 100 | 0 |

**Glycine standard curve**

**Serine standard curve**

**Generation of metastatic KPC-Ganji & Bassel-Luc cells (mKPC-GB-Luc cells)**

PANC-1 cell lines were grown in DMEM containing 10% FBS for 24 hours. The media was then replaced with serum-free DMEM, and the cells were incubated overnight. The conditioned medium was then collected and filtered through a 0.1 μm pore size filter. The filtered medium was used to culture KPC-Luc cells overnight. Then, the KPC-Luc cells were maintained in 10% FBS in DMEM for 36 hours, after which they were replaced with conditioned media (obtained from PANC-1 cell culture medium). They were then kept for an additional 12 hours over 30 days. To validate the metastatic potential, mKPC-GB-Luc cells were orthotopically implanted into mice. After orthotopic implantation, 80% of mice developed liver metastasis, and 100% developed lung metastasis. To elucidate changes in mKPC-GB-Luc cells, we compared bulk RNA sequencing with that of the parent cell line.

**Bulk RNA sequencing**

RNA isolation, purification, and RNA sequencing were performed on six samples, including two groups (KPC-Luc and mKPC-GB-Luc), according to the company's standard protocol (Admera Health, South Plainfield, NJ, USA). In brief, the quality of the extracted RNA sample was checked using a High Sensitivity RNA TapeStation kit (Agilent Technologies Inc., California, USA). The sample concentration was measured using a Qubit 2.0 RNA HS kit (Thermo Fisher, Massachusetts, USA). Paramagnetic beads coated with oligo d(T)25 are used to isolate poly A+ containing RNA using the NEBNext® Poly(A) mRNA Magnetic Isolation Module kit (New England BioLabs Inc., Massachusetts, USA). Before first strand synthesis, random priming using 5’ d(N6) 3’ [N=A, C, G, & T] and fragmentation are carried out as recommended by the manufacturer. First-strand synthesis is carried out using Protoscript II reverse transcriptase with an extended extension time of 30 minutes at 42 °C. All other steps are carried out as recommended by the manufacturer using the NEBNext® Ultra™ II Directional RNA Library Prep Kit for Illumina® kit (New England BioLabs Inc., Massachusetts, USA). The final library concentration is checked using a Qubit 2.0 kit (Thermo Fisher, Massachusetts, USA), and library quality is checked using a TapeStation HSD1000 ScreenTape kit (Agilent Technologies Inc., California, USA). The final library size was approximately 430 bp, with an insert size of approximately 300 bp. Illumina 8 nt dual indices were used. Equimolar pooled libraries were made based on QC values. Libraries were then sequenced using an Illumina 8-nt dual-index with a read length of 150 PE, producing 40M PE reads per sample (20M in each direction). Each group has three biological replicates. The FASTq files were run through nf-core/RNAseq version 3.21.0 with the GRCm38 mouse genome reference. The resultant count matrices from STAR-SALMON output were used for downstream analysis with DESeq2 for differential gene expression, gene ontology, and KEGG pathway analysis.

***In vivo* studies**

Orthotopic pancreatic cancer mice were generated using our previously established protocol [1]. Mice mKPC-GB-Luc (Developed by Ganji and Bassel; RRID: CVCL_UY60) cells (50000 cells/40 μL/animal) and or KPC-ML1-Luc cells (50000 cells/40 μL/animal) were injected into the mid area of the pancreatic region of C57BL/6J mice (Strain#000664; RRID: IMSR_JAX:000664; Jackson Labs). After the injection, the pancreatic leakage was prevented, and the abdomen was stitched with soluble stitches. All mice were monitored daily in accordance with UAB IACUC guidelines (IACUC-22541). The treatment (IP for KPC-GB-Luc and IV for KPC-ML1-Luc) was initiated on day 5 after cell injection. Experimental details were described and depicted in Supplementary Table 2 and Fig. 1C. The FOI dose schedule was chosen according to previous reports of preclinical studies of FOLFIRINOX/FOI regimens and fell into the range of typical doses applied to mice with PDAC [2]. The chosen doses were aimed at delivering the drug combination with clinical relevance and ensuring therapeutic efficacy while maintaining tolerable toxicity levels.

For the mKPC-GB-Luc study, we used 5 animals per group. Group one received PBS and served as a sham; the second group received FOI (Fluorouracil 25mg/Kg, Oxaliplatin 5mg/Kg, Irinotecan 25 mg/Kg) [2] IP twice weekly and ProAgio (10mg/Kg) IP twice weekly and fourth group received ProAgio and FOI. For the KPC-ML1-Luc study, we used 10 animals per group. Animals were treated with PBS (sham) or gemcitabine and nab-paclitaxel (GPTx; G, 25mg/kg; PTx, 5mg/Kg) IV twice weekly, ProAgio (10mg/kg) IV twice weekly, and the fourth group received ProAgio and GPTx. The IVIS system (bioluminescent imaging) was used to monitor tumor progression on days 5 and 29 for mKPC-GB-Luc, and on days 5 and 26 for KPC-ML1-Luc after therapy. At the end of the study, at least 2 hours before euthanasia, pimonidazole (60mg/Kg; notably, the hypoxic probe's detection is unaffected by fixation. Pimonidazole hydrochloride creates protein adducts in hypoxic cells upon *in vivo* administration. This protein adduct can be detected with a specific monoclonal antibody. The detection of pimonidazole is a reliable and direct indicator of tissue hypoxia. Following euthanasia, mKPC-GB-Luc tumors, liver, and lung were collected. Lung and liver tissues were fixed, processed into FFPE blocks. Fifteen representative 6 micrometer sections from each organ were cut at predetermined intervals and mounted on slides. Slides were stained with hematoxylin and eosin (H&E). A pathologist masked to treatment assignment examined lung and liver sections at 4X and 20X magnification (scale bar 50µm) for metastasis. Number of animals in each group with metastatic lesions was recorded. Quantification was performed using GraphPad Prism software. After euthanasia, KPC-ML1-Luc cells' metastasis was analyzed in the liver and lungs using PerkinElmer IVIS Lumina III *In Vivo* Imaging System (RRID:SCR_025239). Tumors were weighed, stored, and processed for further biomarker analysis (flow cytometry, immunohistochemistry, and immunofluorescence).

Rag knockout (KO) experiments were performed in orthotopic pancreatic tumor models, similar to those described above in the initial set of experiments, to assess the overall efficacy of combination regimens (Table 2). Briefly, the first group (n=10) received PBS (IV) and the second group (n=10) received GPTx+ProAgio (IV) twice weekly for 26 days. Doses are given as indicated in the above experiment. At the end of the experiment, the first group had 6 animals, and the second group had 9. Following euthanasia, KPC-ML1-Luc tumors, liver, and lung were collected. After euthanasia, KPC-ML1-Luc cells' metastasis was analyzed in the liver and lungs using PerkinElmer IVIS Lumina III *In Vivo* Imaging System (RRID: SCR_025239). Tumor images were captured, and tumors were weighed to determine the combined effect on tumor progression.

GEM-KPC mice (LSL-KrasG12D/+; LSL-Trp53 R172H/+; Pdx-1-Cre) were obtained from Jackson Laboratory (Bar Harbor, Maine). These mice were created by mating LSL-KrasG12D/+ with LSL-Trp53R172H/+ and then breeding the resulting heterozygotes with Pdx-1-Cre. All mice were monitored daily in accordance with Georgia State University (GSU) IACUC guidelines (IACUC-14028). GEM-KPC mice were selected for survival investigation on a rolling basis, as previously described [3].The GEM-KPC mice were evaluated using ultrasound at 13 weeks of age. Animals with documented tumors were divided into 4 groups and treatment was started on week 14. Experimental details were described and depicted in Supplementary Table 2 and Fig. 4A. Initially, they were treated with ProAgio (10mg/Kg; IP) for 10 consecutive doses, trailed by 10 doses every other day. Gemcitabine (50mg/Kg; IP) treatment was initiated after 5 doses of ProAgio and administered once every 2 days. In this study, the technique of censoring was used for the GEM-KPC mouse model, ensuring that survival analysis results are specifically focused on mortality from PDAC and not from any other non-PDAC-related reasons. The animals were monitored, and if any of them died cause of death was established. The non-PDAC related causes of mortality may include the onset of any non-PDAC-related cancer, such as sarcomas, melanomas, or lymphomas. This also include the onset of severe non-PDAC-related diseases that may require euthanasia, such as prolapse. These animals were censored. In addition, animals that are still alive after a predetermined time were censored. Survival estimates were calculated using the Kaplan-Meier method, with Log-rank (Mantel-Cox) testing of survival curves from each group. The criteria for euthanasia include severe body weight loss, decreased movement, lethargy, abdominal swelling, and poor overall body condition in line with guidelines from the IACUC facility. For the efficacy study, the animals were subjected to the same treatment conditions for 4 weeks. At the end of the experiment, blood was collected, and tumors were isolated from each group and processed for ELISA, IHC, and ICC analysis.

**Supplementary Table 2: Experimental design, treatment, dose,** and mode for animal work.

| **Experiment #; Model; Group #; Animal #** | **Treatment** | **Dose and frequency; Administration route** |
| --- | --- | --- |
| **Experiment 1** |  |  |
| **mKPC-GB-Luc orthotopic C57BL/6J mouse model for analysis** |  |  |
| Group 1 (n=5) | Sham (1%DMSO in PBS) | 3.5mL/Kg, twice weekly; IP |
| Group 2 (n=5) | 5-fluorouracil (5-FU), oxaliplatin, and irinotecan (FOI) | 25mg/Kg, 5 mg/Kg, 25 mg/Kg, twice weekly (altogether); IP |
| Group 3 (n=5) | ProAgio (PA) | 10mg/Kg, twice weekly; IP |
| Group 4 (n=5) | FOI + PA | (25 + 5 + 25 + 10) mg/Kg, twice weekly (altogether); IP |
| **Experiment 2** |  |  |
| **KPC-ML1-Luc C57BL/6J mouse orthotopic model for analysis** |  |  |
| Group 1 (n=10) | Sham (1X PBS) | 3.5mL/Kg, twice weekly; IV |
| Group 2 (n=10) | Gemcitabine, Nab-Paclitaxel (GPTx) | 50 mg/Kg, 5mg/Kg, twice weekly (altogether) IV |
| Group 3 (n=10) | ProAgio (PA) | 10mg/Kg, twice weekly; IV |
| Group 4 (n=10) | GPTx+ PA | (50+5+10) mg/Kg, twice weekly (altogether) IV |
| **Experiment 3** |  |  |
| **KPC-ML1-Luc orthotopic Rag-KO model for analysis** |  |  |
| Group 1 (n=10) | Sham (1X PBS) | 3.5mL/Kg, twice weekly; IV |
| Group 4 (n=10) | GPTx+ PA | (50+5+10) mg/Kg, twice weekly (altogether) IV |
| **Experiment 4**  **GEM-PDAC model for analysis** |  |  |
| Group 1 (n=5) | Sham (1XPBS) | 3.5 mL/ Kg; IP |
| Group 2 (n=5) | Gemcitabine (Gem) | 50 mg/Kg; IP |
| Group 3 (n=5) | ProAgio (PA) | 10 mg/Kg; IP |
| Group 4 (n=6) | Gem + PA | (50 +10)/Kg; (altogether) IP |
| **Experiment 5**  **GEM-PDAC model for survival** |  |  |
| Group 1 (n=15) | Sham (1XPBS) | 3.5 mL/Kg; IP |
| Group 2 (n=14) | Gemcitabine (Gem) | 50 mg/Kg; IP |
| Group 3 (n=15) | ProAgio (PA) | 10 mg/Kg; IP |
| Group 4 (n=19) | Gem + PA | (50 +10)/Kg; (altogether) IP |

**Single cell preparation**

Single cells were produced according to our lab standard protocol [1]. Approximately 100-150mg of tumor slice was collected from five animals in each group and placed in a C-tube containing digesting media (5 ml). The digestion medium consists of DMEM containing 1% FBS, 2,000 U DNase I, 1.5mg/ml collagenase D, and 25 μg/ml Liberase. The tumor fragments were dissociated into single cells twice per tube using a mild MACS dissociator (Miltenyi Biotec), then incubated at 37°C for 15 minutes while being shaken continuously. The single-cell medium was filtered through a 100µm cell strainer into a 50 mL tube. The filtrate cells were rinsed with ice-cold PBS and centrifuged at 575 g for 5 minutes. After three 1X DPBS washes, the cells were re-suspended in 10% FBS in DMEM media for further examination.

**Single cell RNA sequencing (scRNA-seq)**

For scRNA-seq, we used 3 FFPE samples from each group of the GEM-PDAC model, prepared an RNA library, and sequenced according to the standard protocol used at Admera Health (South Plainfield, NJ, USA). FFPE blocks were sectioned. RNA was extracted using the Mag-Bind® FFPE DNA/RNA 96 Kit (Omega, Georgia, USA) via the manufacturer’s protocol. RNA quality was assessed via the Infinite F Nano+ 200 Pro Tecan® (Tecan, Switzerland). After RNA QC, tissue was dissociated, and nuclei were isolated following the sample preparation from FFPE Tissue sections for GEM-X Flex Gene Expression protocol (10x Genomics, CG000784 | Rev A). The quantity and quality of the nuclei were assessed by acridine orange and propidium iodide dye on a Cellometer Auto 2000 (Nexcelom). Probes with unique barcodes were added to each sample for hybridization. After hybridization, samples were pooled in a ratio to recover approximately 10,000 nuclei per sample and loaded on a 10x Chromium X using Chromium fixed RNA profiling reagent kits (10x Genomics). Subsequently, library construction was completed according to the manufacturer's protocol, with 11 PCR cycles. The quality of the final library was assessed using the High Sensitivity D1000 TapeStation (Agilent Technologies Inc., California, USA) and quantified using the Qubit 2.0 DNA HS assay. Libraries were sequenced on an Illumina® NovaSeq X Plus (Illumina, California, USA) with a read length of 150 PE, targeting a total of 800M reads per pool (400M in each direction). The raw scRNA-seq data were demultiplexed, aligned to the mouse reference genome (GRCm39) for gene expression quantification, and preprocessed with Cell Ranger 9.0.1 using the default settings. The output count matrix files were further evaluated and visualized using the Seurat (v.5.3.0) R package (v.4.5.1; RRID: SCR_016341). We filtered based on the following criteria: nFeature_RNA (nGene) > 200, and percent.mt < 5 (5% for mitochondrial genes). After filtering out undesirable cells and mitochondrial genes, the SC transform method was used to normalize the data. Then, we performed data scaling, linear dimensionality reduction (LDR), and cell clustering using PCA and UMAP with default settings.

Using the FindClusters technique, we created 20 clusters from the top 30 principal components (PCs) at a resolution of 0.3. The default FindAllMarkers method was used to annotate each cluster's differentially expressed genes (DEGs) with positive and negative markers, and the Wilcoxon rank-sum test was then applied. We also confirmed the clusters using well-known canonical gene markers for Granulocytic MDSCs, Myocytes, Dendritic cells, T, NK, and B cells, endothelial cells, fibroblasts, acinar-to-ductal metaplasia (ADM), and PDAC cells.

Finally, we allocated suitable cell type names to each cluster. Furthermore, we were interested in a subgroup of fibroblasts, macrophages, and T cells. These cells were subsetted and standardized; highly variable genes were identified; the data were scaled; and dimensionality reduction was performed using the Seurat R package.

**Flow cytometry analysis**

Sample preparation and analysis followed the procedures reported in earlier investigations [1]. Red blood cells were removed from single-cell media using ACK lysis buffer. The cleaned cells were immersed in the Fc-blocking reagent. Then, the single cells were exposed to a live/dead stain for 15 minutes, followed by the addition of primary fluorophore-conjugated antibodies for 60 minutes on ice. They were then washed twice with 2% BSA in 1X DPBS, with a 5-minute interval between washes. Following washing, the cells were stained for surface antigens in the dark and at RT. BD GolgiPlug and BD leukocyte activation cocktail (BDB550583) stimulated leukocytes. After permeabilization, the eBioscience™ Foxp3 kit (Catalog no. 00-5523-00) was used for intracellular staining in the dark. Stained cells were fixed with buffered paraformaldehyde and filtered before running the flow (FACSymphony A5). The flow data was analyzed using FLOWJO software (version 10.7.2; RRID: SCR_008520; Becton Dickinson).

**Immunohistochemistry (IHC) and immunofluorescence (IF)**

The IHC and IF staining procedures were performed according to our lab standard protocol. Reagent and kit details were provided in Supplementary Table 1. Images were captured at 20X magnification, and quantification was performed using ImageJ software.

**Multiplex Immunofluorescence (COMET)**

*Tissue microarray design (TMA):* TMA was constructed using murine tumors harvested from GEMM PDAC (KPC) tumors. It consisted of 12 cores arranged in a 3×4 grid (11 tissue cores + 1 empty position at C2). Sham (n=5, cores A1, B1, C1, D1, D2) and Gem+ProAgio treated group (n=6, Cores A2, A3, B2, B3, C3, D3). A bubble artefact was detected in core B2, obscuring 25-30% of the lower-left half of the tissue, and this region was excluded from the analysis.

*Multiplexed immunofluorescence staining*: Staining on TMA was performed using Lunapore COMET platform with a 40+ marker panel in two subsequent imaging runs. Markers in the panel are listed in Supplementary Table 3. Part 1 run consisted DAPI, CD31, CD11c, FoxP3, NCR1, CD3, Cleaved Caspase 3, PD-1, E-Cadherin, CD8, podoplanin, CD4, CTLA-4, MHC-II, Cd45, Cd19, Ly6C, T-bet, αSMA, Ly6G, XCR1, Cd69, VEGF-A, Granzyme B, IL-6, PDGFR-β, Integrin-β3, CD172α, IFN-γ, TGF- β, FAP, autofluorescence reference channels. Part 2 run consisted of DAPI, CD11b, pan-keratin, MHC-I, CD206, Ki-67, F4/80, keratin17/19, vimentin, collagen 1, and reference channels.

*Image registration*: Both the Part 1 and Part 2 images were captured in OME-TIFF file format. Part 1 and Part 2 were aligned by performing phase cross-correlation on the DAPI channel, followed by affine transformation alignment. No rescaling was performed because both parts had identical pixel resolutions (0.23 μm/pixel).

*TMA core detection*: TMA cores were detected by down-sampling the DAPI channel to 8x, following which a Gaussian blur (σ =50) was applied with Otsu’s thresholding, connected component analysis was performed, and morphological clean-up was performed. Detected centroids were then assigned positions (A1-D3) utilizing Agglomerative clustering.

*Cell segmentation*: Cellpose v4.0 (nuclei model) was used to perform cell segmentation with the following parameters: diameter = 8.0 um / 0.23 um/pixel (approximately 35 pixels), cell probability threshold = -1.0, and flow threshold = 0.4. Furthermore, the mask of the nuclei was expanded by 3 µm using skimage.segmentation.expand_labels. 399,268 cells were detected and segmented across 11 cores.

*Compartment classification and immune cell phenotyping*: The tissue was classified into three compartments: Neoplastic (Pan-Keratin^+^ or Keratin 17/19^+^ and CD45^-^), Immune cells (CD45^+^), and stromal compartment (Pan-Keratin^-^ and Keratin 17/19^-^ and CD45^-^). T cells – CD4 T cell (F4/80^-^, CD4^+^), CD8 (F4/80^-^, CD8^+^), T regs (CD4^+^, FoxP3^+^ F4/80^+^). Macrophages(F4/80^+^), M1 macrophage (F4/80^+^, MHC-II^+^), M2 macrophage (F4/80^+^, CD206, MHC-II^-^), M1/M2 double positive (F4/80^+^, MHC-II^+^, CD206^+^), B cells (CD19^+^), NK cells (NCR1^+^), Dendritic cells (CD11c^+^). For functional state analysis of CD8 and CD4 T cells, the following markers were used: T-bet (Th1 transcription factor), PD-1 and CTLA-4 (exhaustion and checkpoint), Granzyme B (cytotoxicity), IFN-γ (effector function), and Ki-67(proliferation). Endothelial cells (CD31^+^), myCAFs (FAP^+^ or Podoplanin^+^ and αSMA^+^), iCAF (αSMA^-^, Ly6C^+^ or IL-6^+^), apCAF (CAF marker^+^ MHC-II^+^ αSMA^-^), qCAF (FAP (low), Podoplanin (low) Vimentin^+^, αSMA^-^, MHC-II^-^, IL-6^-^, and Ly6C^-^). Moffitt’s classification of tumor cells includes Classical (E-cadherin high), Basal (Keratin 17/19 high or vimentin high and E-cadherin low), and unclassified (neither classical nor Basal criteria are met).

*Statistical analysis:* scipy.spatialcKDTree was used to calculate nearest neighbor distances. The Euclidean distance for the nearest cell of interest was calculated in physical units (μm). Mean distances were compared across treatment groups for each individual core. By identifying cells within 20μm and 50μm radii of each cell of interest, the cellular neighborhood was determined, after which proportions were calculated for each compartment among neighbors. A3 and C3 cores were excluded from the spatial analysis due to low immune cell counts. Several different approaches were used in the statistical analysis of the data.

*Approach 1*: Pooled cell analysis – Cumulatively, all cells of a particular type were pooled across cores within each treatment group. Chi-squared test of independence was utilized to compare the proportion of marker-positive cells across treatment groups, and Fisher’s exact test was used when any expected count was <5. This approach treats each cell as a unit of analysis, thereby increasing statistical power. However, cells present within the same TMA core are not statistically independent (pseudoreplication). This approach may overestimate significance. Per-Core analysis is provided alongside pooled analysis for full transparency.

*Approach 2*: Per-Core Analysis – The proportion of each cell type for each TMA core was determined as a percentage of the parent population. Each core represents a biological replicate. A two-sided Mann-Whitney U test (scipy.stats.mannwhitneyu) was used to compare proportions between the treatment groups.

*Approach 3*: Pooled spatial Distributions – Kolmogorov-Smirnov (KS) two-sample test was performed on pooled distance distributions across treatment groups. Additionally, a chi-squared test on pooled counts was performed to assess the proportions of cells within 20 μm of the nearest tumor cells.

*Data presentation*: Data are presented as bar graphs, with pooled proportions (weighted by cell count) shown as bar heights. Error bars represent the standard error of the mean calculated per-core proportions. Each bar shows data points representing per-core proportions. Significance: *** *p*<0.001, ** *p*<0.01, * *p*<0.05, ns = not significant.

**ELISA**

This was performed according to the R&D protocol to quantify the protein expression in serum and tumor samples. Respective antibody-coated 96-well plates were obtained from the appropriate kit. The standards and experimental samples (serum or tumor supernatant) were added to the plate and incubated for 1 hr in the dark at RT. After incubation, the plate was washed three times with washing buffer, with a 5-minute interval between washes, to remove unbound proteins. Then add HRP-conjugated detection antibody to each well and incubate for 45 minutes. After incubation, wash the plate three times with washing buffer, then add the Tetramethylbenzidine (TMB) substrate solution to each well. A blue color was developed in proportion to the target present in each sample. A reaction-stopping solution was added to each well, and the wells turned yellow. Finally, the absorbance at 450 nm was measured, yielding a signal proportional to the amount of target bound to the respective antibody.

**Quantification and statistical analysis**

A one-way ANOVA, succeeded by Tukey’s test for pairwise comparisons, was conducted for multiple groups' mean comparisons (GraphPad Prism 10.0.2; RRID # SCR_002798). In the absence of parametric test assumptions, a nonparametric test, such as the Kruskal-Wallis test (for three or more groups), was employed. Results were presented as mean ± standard error. Unless otherwise specified, every test was two-sided. G-power 3.1.9 (RRID: SCR_013726) was used to calculate the sample size for a power of 0.8 at a p-value of 0.05. Unpaired t-tests with Welch's correction were used to compare sham and ProAgio groups (IP and IV) for continuous variables. For Liver and Lung mets, pairwise group comparisons were performed using Fisher’s exact test. To compare trends across ordered groups, the Cochran-Armitage trend test was employed.

**References**

1.	Nagaraju GP, Saddala MS, Foote JB, Khaliq AM, Masood A, Golivi Y, Bandi DSR, Sarvesh S, Reddy SP, Switchenko J: **Mechanism of enhancing chemotherapy efficacy in pancreatic ductal adenocarcinoma with paricalcitol and hydroxychloroquine.** *Cell Reports Medicine* 2025, **6**.

2.	Carotenuto P, Amato F, Lampis A, Rae C, Hedayat S, Previdi MC, Zito D, Raj M, Guzzardo V, Sclafani F: **Modulation of pancreatic cancer cell sensitivity to FOLFIRINOX through microRNA-mediated regulation of DNA damage.** *Nature communications* 2021, **12:**6738.

3.	Uddin MH, Al‐Hallak MN, Khan HY, Aboukameel A, Li Y, Bannoura SF, Dyson G, Kim S, Mzannar Y, Azar I: **Molecular analysis of XPO1 inhibitor and gemcitabine–nab‐paclitaxel combination in KPC pancreatic cancer mouse model.** *Clinical and Translational Medicine* 2023, **13:**e1513.
